# Whole Cells as Biocatalysts in Organic Transformations

**DOI:** 10.3390/molecules23061265

**Published:** 2018-05-25

**Authors:** Fabián Garzón-Posse, Liliana Becerra-Figueroa, José Hernández-Arias, Diego Gamba-Sánchez

**Affiliations:** Laboratory of Organic Synthesis Bio- and Organocatalysis, Chemistry Department, Universidad de los Andes, Cra. 1No 18A-12 Q:305, Bogotá 111711, Colombia; fa.garzon10@uniandes.edu.co (F.G.-P.); lm.becerra84@uniandes.edu.co (L.B.-F.); jm.hernandez2@uniandes.edu.co (J.H.-A.)

**Keywords:** biocatalysis, whole cells, enantioselective transformations

## Abstract

Currently, the power and usefulness of biocatalysis in organic synthesis is undeniable, mainly due to the very high enantiomeric excess reached using enzymes, in an attempt to emulate natural processes. However, the use of isolated enzymes has some significant drawbacks, the most important of which is cost. The use of whole cells has emerged as a useful strategy with several advantages over isolated enzymes; for this reason, modern research in this field is increasing, and various reports have been published recently. This review surveys the most recent developments in the enantioselective reduction of carbon-carbon double bonds and prochiral ketones and the oxidation of prochiral sulfides using whole cells as biocatalytic systems.

## 1. Introduction

The enantioselective synthesis of organic compounds is one of the greatest challenges in organic chemistry, mainly because of its importance in the development of compounds with biological activity as current drugs or potential new drugs. With this aim, several catalytic processes have been reported, such as reactions with transition metal catalysts, organic catalysts and biological catalysts. The latter is the focus of this manuscript.

Biocatalysis is the use of isolated enzymes or whole cells (bacteria, fungi, microalgae and plants, among others) as catalysts in organic reactions [1,2]. This synthetic strategy often provides high enantioselectivity; additionally, other advantages such as benign reaction conditions, low toxicity, the possibility of recycling and the production of eco-friendly waste make biocatalysis one of the most important tools to create new stereogenic centers and the perfect “green” technique [3,4].

Most publications on biocatalysis focus on isolated enzymes obtained by the overexpression of enzymes in genetically engineered microorganisms; however, the use of these biocatalysts is expensive and requires special techniques and resources, such as auxiliary enzymes (increasing the cost) to renew the cofactors or cloning techniques, respectively [5,6]. Even if these approaches are important alternatives, they are not commonly used in laboratories investigating organic synthesis. The use of whole cells as biocatalysts in several enantioselective reactions has been reported, and, recently, new reports have been published on this topic, showing that it is a simpler and equally effective method to obtain high yields and enantiomeric excess (e.e.) in reactions designed to synthesize biologically active compounds [7,8]. Furthermore, studies with whole cells are generally the first step in the search for new enzymes with applications in enantioselective transformations. Despite the substantial advances, new research is still needed to overcome some significant drawbacks, such as substrate/product toxicity, or to scale up the reactions.

Some particular aspects of the use of whole cells to develop efficient applications in practical organic biotransformations, such as the strategies applied to design and optimize the organisms modified for chemical production [9], the current progress in the development of immobilized whole-cell biocatalysts, the immobilization methods and the bioreaction engineering [10], and the toxic solvent properties of ionic liquid/water systems [11], have recently been reviewed. This review surveys recent developments in biocatalysis using whole cells; it begins with a presentation of the evolution of the concept in the first section, followed by a detailed description of applications in asymmetric reactions, such as the reduction of C=C bonds, reduction of prochiral ketones, oxidation of sulfides to chiral sulfoxides, and finally, some other less common transformations, while focusing the developments reported in the last ten years.

## 2. Evolution of the Concept

Notably, humans have long been interested in the power of natural systems to perform chemical transformations; the evolution of metabolic processes has been studied by biologists, microbiologists, and biochemists for many years. Therefore, the knowledge about enzymes and the chemical reactions they perform is continuously increasing and other scientists are able to use that knowledge in their own fields. A perfect example is the bridge between biocatalysis and organic synthesis.

The discipline of synthetic organic chemistry has focused its efforts on five main targets: (i) the development of new chemical reactions; (ii) improvements in chemo and regioselectivity; (iii) improvements in reaction yields; (iv) the identification of milder reaction conditions, which have become particularly important in recent years with the birth of the “green chemistry” concept; and finally (v) the development of enantioselective chemical transformations. On the other hand, biocatalysis, a tool used in organic chemistry laboratories, combines two concepts of enzymatic transformations: first, the use of selected enzymes to perform specific organic reactions, even with polyfunctional substrates, and second, the use of several enzymes in cascades, emulating natural metabolic processes under normal conditions. That fusion of concepts summarizes the evolution of biocatalysis in organic synthesis.

Some authors have described the above mentioned evolution as three waves of biocatalysis [12,13,14]. The first modern evidence revealing the power of nature to produce enantiopure compounds was the work of Louis Pasteur in 1848 [15]; then, chemists understood that some components in living cells were capable of performing exogenous organic transformations. These findings represent the first wave of biocatalysis, which is based on the use of enzymes or whole cells in some chemical reactions with the aim of producing attractive metabolites. The second wave tried to extend the range of substrates for particular enzymes to promote the synthesis of uncommon products; during this second wave, studies to improve the stability and recovery of the enzymes were performed, some of which are still useful and compatible with more recent developments. Finally, the third wave focused on engineering enzymes to fit the desired process, namely, new enzymes were used to perform non-natural reactions in various pathways.

In our opinion, the outstanding influence of biocatalysis on organic synthesis is explained by the mixed use of those three waves or three approaches in the laboratory; as the reader will notice in this review, the five targets of synthetic organic chemistry are reached using biocatalysis. Additionally, as mentioned above, the birth of green chemistry concepts prompted organic chemists to think about new eco-friendly methods, and biocatalytic processes are by far the ideal processes to fit green chemistry principles.

In summary, whether through the use of isolated enzymes (natural or designed) or through the use of whole cells of microorganisms, vegetables or even cultures of genetically modified cells, the use of biocatalysis has wonderful applications in synthetic organic chemistry.

## 3. Reduction of C=C Bonds

Chemical transformations mediated by biocatalysts are very attractive alternatives to the standard chemical methodologies for the preparation of elaborate organic substrates and chiral compounds. The most frequent methodologies for reducing carbon-carbon double bonds exploit non-selective chemical methods with inappropriate conditions for the environment; in contrast, the use of biocatalysis is more often characterized by mild reaction conditions, eco-friendly procedures and high selectivity (chemo-, regio- and stereoselectivity). As a result, biocatalytic processes have emerged as sustainable techniques in organic synthesis because of their multiple advantages [16]. Several applications of the bioreduction of double bonds are presented throughout this chapter. Before focusing on enantioselective bioreduction, we will describe some fundamental developments that may be applied in more complex systems. Even if these developments are exemplified by the formation of achiral products, they include free cells and supported processes, as well as novel biphasic methodologies used to screen for biocatalysts and to reduce C=C double bonds activated by two electron-withdrawing groups. Next, several selected examples of diastereoselective and enantioselective biocatalyzed reduction reactions with significant applications in total synthesis and the production of fine chemicals are presented.

Isolated enzymes and whole cells are being used as biocatalysts in asymmetric synthesis. However, while the use of the former is commonly associated with a greater to maintain catalytic activity enantiomeric excess and excellent chemoselectivity, reduced cofactors must be regenerated in situ using a second catalytic cycle or must be provided in stoichiometric amounts to maintain the catalytic activity. On the other hand, the use of whole cells takes advantage of their own cofactor regeneration system and does not require exogenous cofactors.

**Scheme 1:**
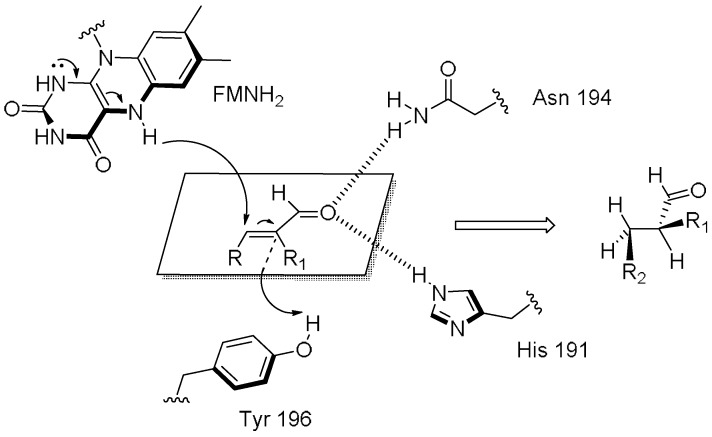
Biocatalyzed C=C double bond reduction mechanism.

Biocatalytic hydrogenation of alkenes to chiral compounds has become an equally useful strategy compared to metal-assisted hydrogenation reactions. The bioreduction of isolated C=C double bonds is a highly desirable transformation. Nevertheless, to the best of our knowledge, no enzyme that is able to perform this reaction has been isolated or characterized. Nature has developed a few strategies that allow this reaction to occur; however, all of them are based on several different chemical process, commonly involving the functionalization of an adjacent carbon atom before the concrete reduction occur [17]. In stark contrast, the biocatalyzed reduction of electron-poor C=C double bonds, namely, bonds conjugated to an electron-withdrawing group, is overly widespread in all kinds of organisms and is the central point of this section. This biotransformation reaction is described as the nucleophilic addition of a hydride derived from the flavin cofactor (FMNH_2_) to a C=C double bond via a Michael type reaction catalyzed by enoate reductases (EREDs) [18]. Specifically, the commonly accepted mechanism is based on the addition of the hydride from FMNH_2_ to C(β), followed by the protonation (by the hydroxyl group of a tyrosine residue) of the resulting enolate. Consequently, the double hydrogen bond between the carbonyl group of the substrate and the NH groups of two amino acid residues is crucial for the electronic activation of the double bond and for the orientation of the substrate in the catalytic site of the enzyme. This type of enzyme exclusively reduces conjugated alkenes with electron-withdrawing groups that are also hydrogen bond acceptors (see Scheme 1).

**Scheme 2:**
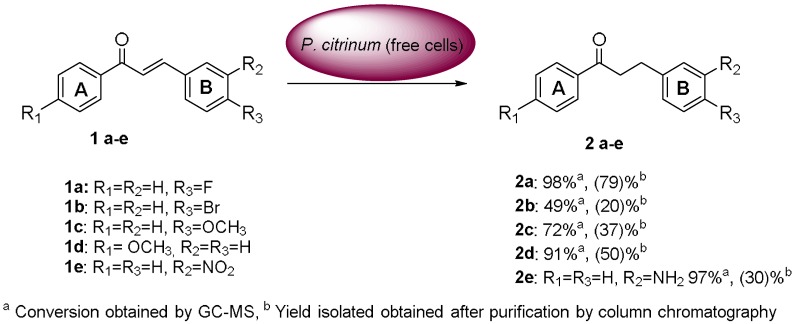
Bioreduction reactions of chalcones using whole free cells of *P. citrinum* CBMAI 1186.

In 2014, Ferreira et al. [19] described the use of whole mycelia of the marine fungus *Penicillium citrinum* CBMAI 1186 free supported on three natural matrices (cotton, fibroin and kapok) to catalyze the reduction of chalcones **1a**–**e** to hydrochalcones **2a**–**e** by fungal enoate reductases in the presence of 50 mg of substrate, with good to excellent yields (Scheme 2). Immobilization of the biocatalyst often implies advantages such as improved process control, increased stability, facilitating the reuse of the biocatalyst and allows the use of reactors with a variety of configurations, among others [20]. Both the immobilized fungus and the free whole mycelium exhibited similar behaviors in the conversion of the chalcones and, in contrast to the free mycelia, the hyphae immobilized on biopolymers were active in biotransforming the substrates after being preserved for 30 days at low temperatures. Moreover, as indicated by the scanning electron micrographs, the cells were intertwined with the fibers of the supports, allowing more efficient separation from the reaction media.

Later, the same researchers tested the ability of whole mycelia to grow in biphasic mixtures containing organic solvents (acetone, ethyl acetate, *n*-butanol, dichloromethane, *n*-hexane and toluene) to broaden the range of applicability of the reduction reaction mediated by the marine fungus *P. citrinum* CBMAI 1186 [21]. The authors employed this strategy to overcome a disadvantage of using biotransformation as a tool for organic synthesis, which is the low solubility of most organic compounds in aqueous media.

**Scheme 3:**
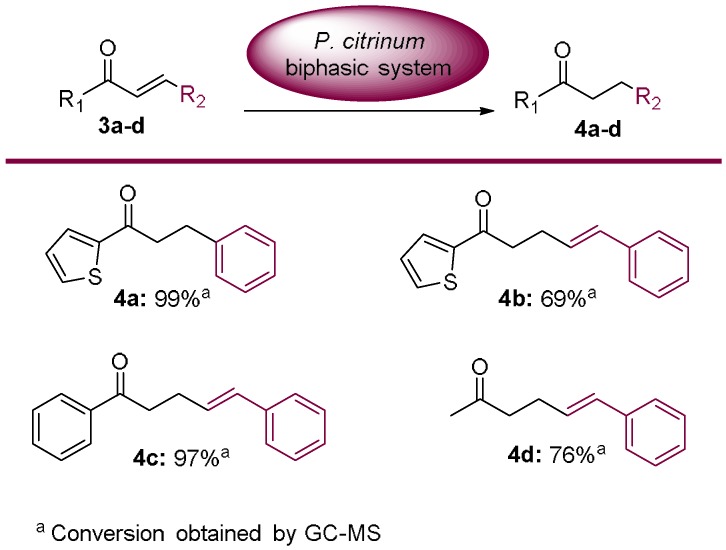
Bioreduction of ketones with the mycelium of *P. citrinum* CBMAI 1186 in a biphasic system.

According to their findings, *n*-hexane was the least toxic solvent, as evidenced by the amount of mycelial mass grown in an artificial seawater medium mixed with each solvent. Thus, the use of whole hyphae as the biocatalyst afforded high conversion rates for the chemoselective biotransformation of the carbon-carbon double bond in α,β-unsaturated ketones (**3**) using a biphasic system of phosphate buffer and *n*-hexane (9:1) on 50 mg scale. Notably, the chemoselective bioreduction of systems with α,β- and γ,δ-unsaturated bonds (substrates **3b**–**3d**) was achieved with excellent conversion; This finding is very representative of these reactions, mainly because this kind of chemoselective reduction is difficult to achieve using conventional nucleophilic hydride sources (Scheme 3).

**Scheme 4:**
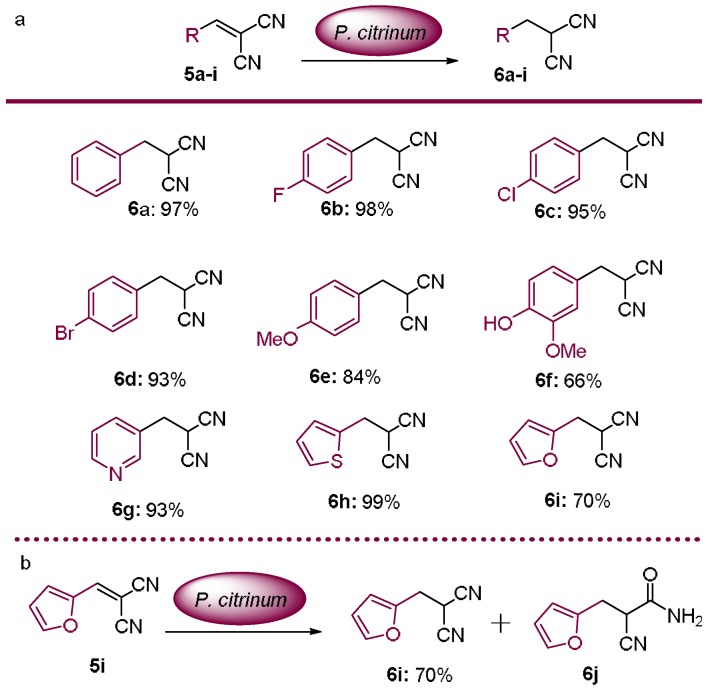
(**a**) Bioreduction of aromatic malononitriles by mycelium of the fungus *P. citrinum* CBMAI 1186; (**b**) hydration of nitrile to amide.

A third communication focused on the biocatalytic reduction of aromatic malononitriles (**5**), which was also promoted by whole cells of *P. citrinum* CBMAI 1186 [22], outlined the versatility of chemoselective biohydrogenation with this fungus. The authors examined the bioreduction of C=C double bonds of different aromatic malononitriles with electron-donating (EDG) and electron-withdrawing (EWG) substituents using 50 mg of starting material. The bioreduction of substrates bearing an EWG in the aromatic ring, such as halogens, was performed and achieved excellent yields; similarly, the bioreduction of aromatic malononitriles with EDG was also achieved, but the yields were slightly lower (Scheme 4a). The authors simultaneously described the hydration of one of the substrates yielding the adduct **6j**, which is the corresponding amide of the nitrile **5j**, under the same reaction conditions. The authors proposed that compound **6j** is the product corresponding to the action of enoate reductases and nitrile hydratase enzymes; see Scheme 4b. This example shows the ability of the biocatalyst to incorporate substrates into potentially useful reaction cascades; unfortunately, data about the enantiomeric excess for the hydration process are not available. We will describe some enzymatic cascades later in this chapter.

**Scheme 5:**
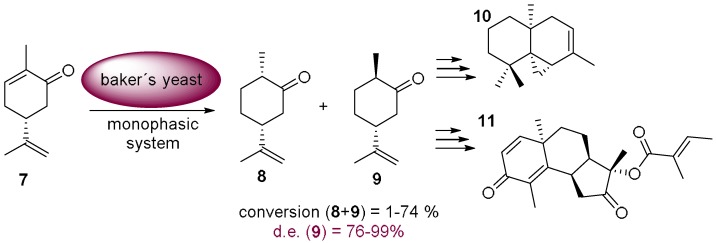
Biotransformation of (4*R*)-(−)-carvone catalyzed by baker’s yeast.

The economic potential for the food, pharmaceutical and perfume industries of products obtained by the microbial and enzymatic transformation of widely available monoterpenoids, such as carvone, menthol, and geraniol, makes this topic a top priority. Recently, Nascimento and coworkers [23] reported the use of commercially available baker’s yeast (BY) as a biocatalyst in the diastereoselective reduction of (4*R*)-(−)-carvone (**7**) in aqueous monophasic or aqueous/organic biphasic systems to obtain (1*R*,4*R*)-dihydrocarvone (**9**) with good conversion and excellent diastereomeric excess (d.e.) on a scale of 100 mg. This compound has been used as a precursor for the synthesis of some molecules of biological interest, such as (−)-thujopsene (**10**) [24] or (+)-decipienin (**11**) [25], as illustrated in Scheme 5.

**Scheme 6:**
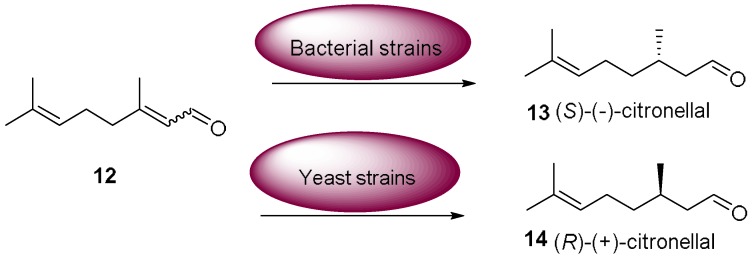
Biotransformation of citral (neral and geranial) into citronellal.

In the same context of the economic exploitation of natural products, specifically for the food and pharmaceutical industries, Rosche et al. [26] tested 20 yeast strains, 9 strains of filamentous fungi and 17 bacterial strains in aqueous systems and in aqueous/organic biphasic systems for their abilities to perform the enantiospecific reduction of the α, β-unsaturated carbon-carbon bond in citral (**12**) to produce citronellal (**13** and **14**). In the traditional aqueous screen, only one bacterial strain was identified as an auspicious biocatalyst; however, the expected reduced product was detected in an aqueous/organic two liquid phase in 11 of the 46 tested strains, clearly showing the benefits of applying two-phase systems in screening strategies. One advantage of this approach is the enhanced solubility of hydrophobic substrates and/or products. The use of a separate organic phase allows high overall concentrations of toxic or inhibitory substrates and products in the reactor while low levels are present in the aqueous phase. Additionally, the permeabilization of the cell membranes is another potential profit of organic solvents. In the same way, in this study, the bioactive bacterial strains preferentially produced the (*S*)-enantiomer of citronellal (**13**), with e.e. values as high as 99%. On the other hand, the eukaryotic strains showed opposite enantiospecificity for the (*R*)-enantiomer (**14**) with an e.e. higher than 98% (See Scheme 6).

**Scheme 7:**
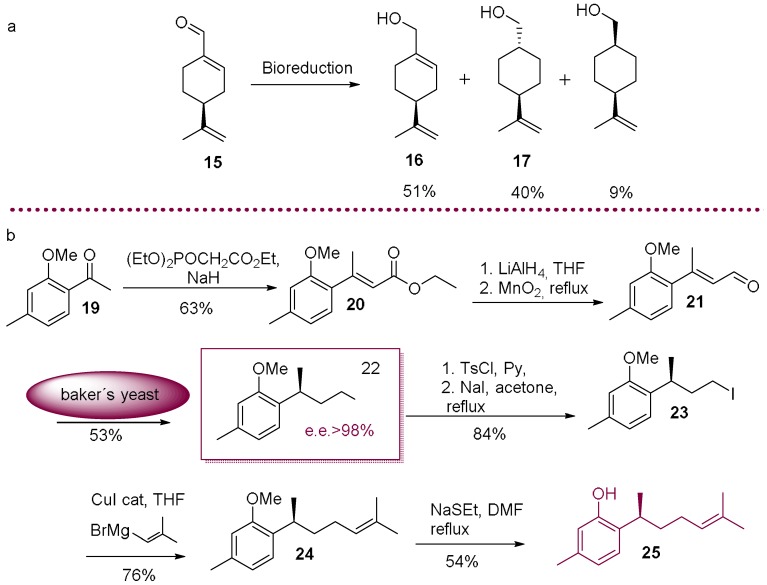

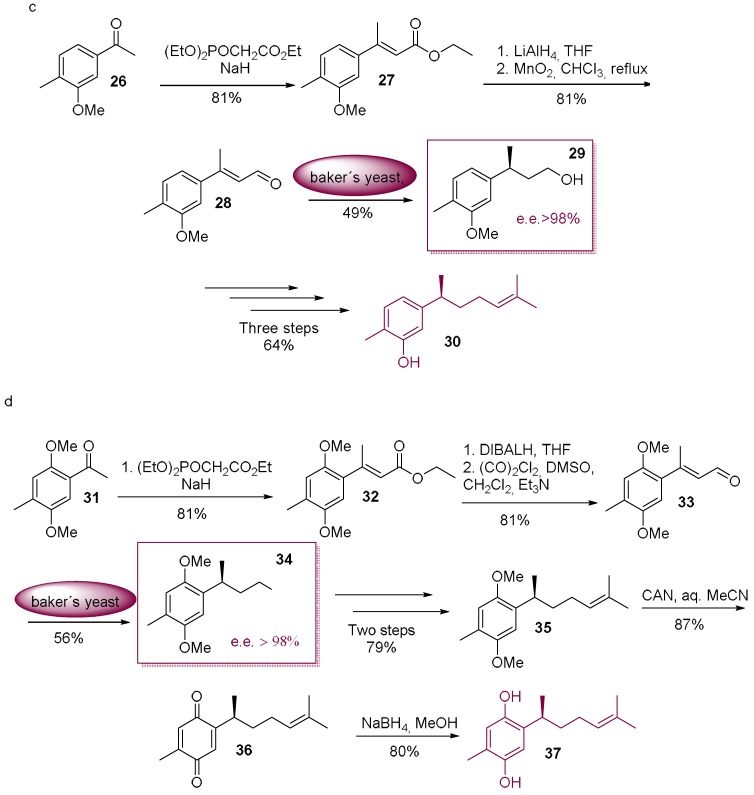
(**a**) Reduction of (*S*)-perillaldehyde; (**b**–**d**) Enantioselective reduction of phenolic bisabolane sesquiterpenes.

As an example of the application of biocatalyzed chiral reduction reactions for the synthesis of complex substances, Serra and coworkers [27] reported the enantioselective synthesis of several phenolic bisabolane sesquiterpenes based on the decagram scale BY-mediated reduction of the corresponding aldehydes to afford important intermediate alcohols **22**, **29** and **34**, which are useful building blocks for the synthesis of phenolic sesquiterpenes with the (*S*) absolute configuration, **25**, **30** and **37**, respectively. These sesquiterpenes of the bisabolane family show a wide range of biological properties, such as antibacterial and antitumor activities. The authors achieved the synthesis of the aldehydes using a common pathway from the related esters through reduction and subsequent allylic oxidation; the esters in turn were synthesized from the corresponding acetophenones via a Horner-Wadsworth-Emmons reaction. The results of the biotransformation showed a conversion of the aldehydes into the saturated alcohols (49–56% yield as isolated products) after only 4–6 days of incubation, with e.e. values of greater than 98%. These results correlated with the findings reported by the same group [28,29], who previously published studies on the regioselective bioreduction of the conjugated double bond of (*S*)-perillaldehyde (**15**), finding that *Euglena gracilis-* and baker´s yeast-mediated biotransformation of this compound produced a mixture of unsaturated and saturated alcohols **16**–**18** (see Scheme 7a).

**Scheme 8:**
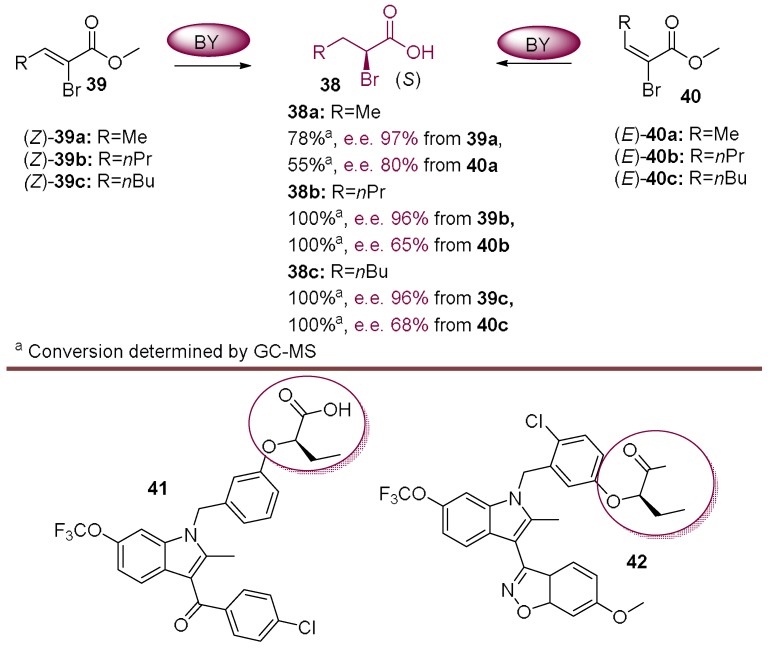
Biocatalyzed reduction of (*E*) and (*Z*) mediated by baker’s yeast. Chiral API incorporating the stereogenic unit are shown.

The transformation of the aforementioned alcohols to the sesquiterpenes of interest was completed by converting these compounds into the related iodides. The coupling of the iodides with the Grignard reagent in presence of copper iodide afforded the corresponding alkenes, and the subsequent removal of the methyl ether functionality afforded the expected compounds (see Scheme 7b–d). Brenna et al. [30] developed a biocatalyzed approach to the synthesis of (2*S*)-bromobutanoic acid (**38**) using BY-mediated fermentation, with an e.e. of greater than 97%. This molecule is an important chiral moiety in the molecular skeleton of a certain class of chiral drugs used for the treatment of non-insulin-dependent type 2 diabetes mellitus (T2DM), **41** and **42**, and it is also commonly referred as an active pharmaceutical ingredient (API) (see Scheme 8).

**Scheme 9:**
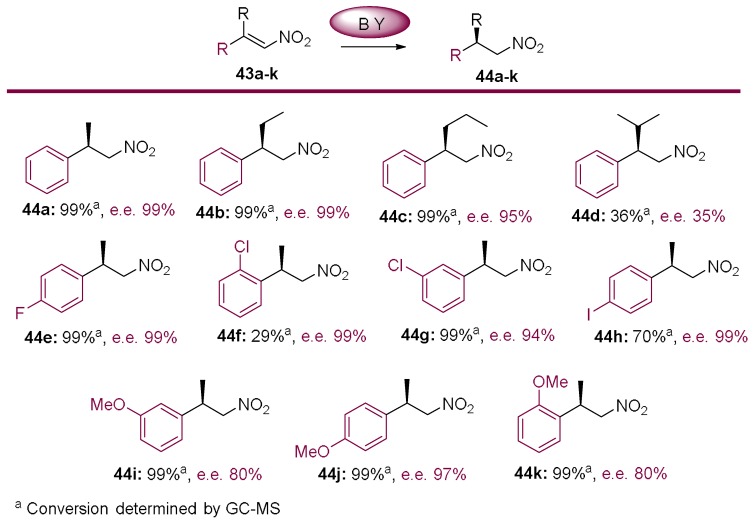
Biotransformation of nitroalkenes.

Recently, the same group published a series of papers focused on the bioreduction of nitroalkene substrates. One of those papers [31] specifically investigated the scope of substrates for EREDs and BY in the enantioselective bioreduction of β-alkyl-β-nitroalkenes (Scheme 9). The enantiomerically enriched products of that transformation reaction are important intermediates in organic synthesis because of the possibility of converting nitro derivatives into amino compounds by reduction [32], carboxylic acids by Meyer reactions [33], aldehydes or ketones by Nef reactions [34], etc. High enantiomeric excess was reported for the reduction of the substrates (**43**), obtaining the (*R*) enantiomer using BY or the (*S*) enantiomer using *Zymomonas mobilis.* The authors also evaluated the effect of sterically and electronically different substituents on the conversion and the enantioselectivity of the biotransformation reaction with BY using a set of nitroalkenes with the substituent on the aromatic ring or on the β-carbon with respect to the nitro group. The authors identified a wide range of accepted substrates; specifically, the presence of a *para* substituent on the aromatic ring, either a methoxy group or a halogen atom, does not exert negative effects on the reaction. 

On the other hand, the *ortho* and *meta* positions may suffer from steric problems due to the clash between the substituents and hydrophobic residues in the active site. Moreover, the steric hindrance of the alkyl substituent on the double bond becomes crucial only when a ramification is present (see compounds **44c** and **44d**). The results (conversion and e.e.) obtained using isolated enzymes and whole cells of BY were virtually the same. The scale of these procedures was approximately 3 g of organic substrate.

**Scheme 10:**
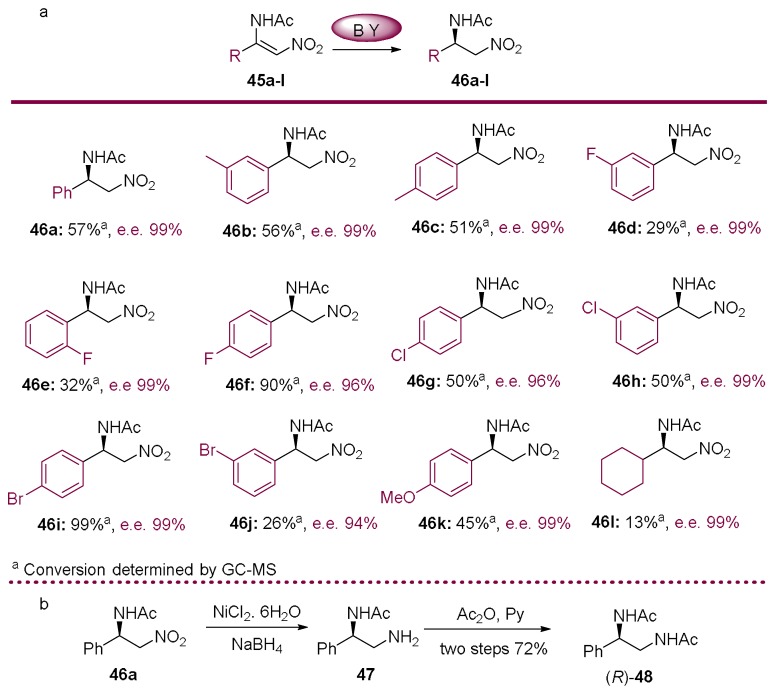
(**a**) Biocatalyzed reduction of β-acylaminonitroalkenes; (**b**) reduction of nitro group into a primary amine.

The second contribution of the Brenna group was the biocatalytic hydrogenation of β-acylaminonitroalkenes (**45**) and the further manipulation of the reduced products [35]. The authors reported higher yields of the isolated products from the bioreduction reaction promoted by isolated enzymes than those obtained with BY, arguing that isolation was difficult; however, the enantioselectivity of both methods was excellent and essentially equal for the 50 mg scale. The electronic properties of the substituents on the aromatic ring had insignificant influences on the outcome of the reaction, and the (*R*) enantiomer of the reduced compound was always obtained; see Scheme 10a. Next, the authors described the synthesis of vicinal diamine functionalities, which are important in active biomolecules and as pharmaceutical ingredients, by converting the nitro group into a primary amine using a reduction–acetylation sequence see Scheme 10b.

**Scheme 11:**
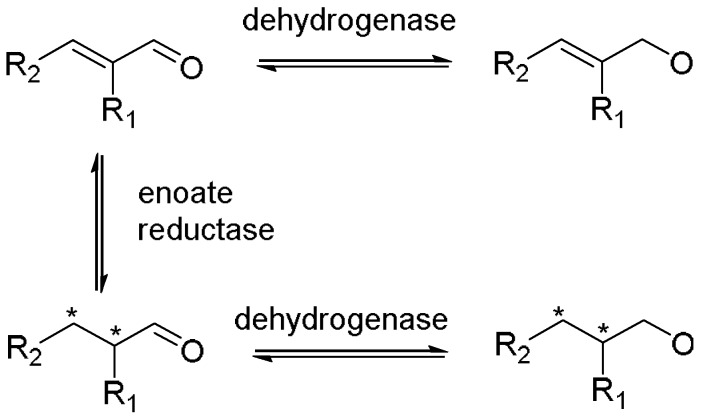
Mechanism for the bioreduction of enals.

Despite the great impact of bioreduction reactions for the synthesis of chiral carbon centers, this procedure has certain limitations when it is used to prepare chiral primary alcohols, mainly due to the considerably competitive formation of allylic alcohols (see Scheme 11).

The conversion of saturated aldehydes into primary alcohols is a fast transformation; as a result, the product distribution depends on the relative reduction rate of the C=C and C=O bonds of enals. The key factor determining the amount of saturated alcohol in equilibrium is the rate of reverse transformation from the allyl alcohol to the enal. 

**Scheme 12:**
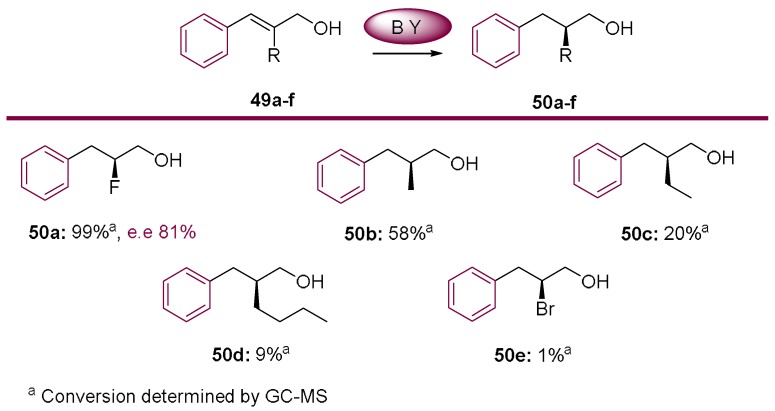
Baker’s yeast-mediated reduction of cinnamyl alcohols.

With the aim of developing an efficient synthetic pathway to produce optically pure fluorinated primary alcohols, Gong et al. [36] studied the 50 mg scale BY-mediated enantioselective synthesis of 2-substituted alcohols **50** through the biocatalyzed reduction of 2-substituted cinnamyl alcohols **49**, initially evaluating the steric effect of several α substituents. As the size of the R group increased, the conversion rate of the biocatalytic process sharply decreased; the authors also found that the process was sensitive to electronic effects. Thus, the inhibitory effect of halogen atoms was attributed to their high negative inductive effect that decreased the electron density on the C=C double bond. Consequently, the poor reactivity of the brominated substrate might be due to the combination of the bromine atom size and electronegativity; on the other hand, the presence of a smaller fluorine atom allowed the reaction to proceed. The researchers found that an increase in the reduction time and optimization of the reaction conditions (pH = 7 and biocatalyst ratio) for the bioreduction of 2-fluorocinnamyl alcohols using baker’s yeast is a convenient way to generate a chiral center containing a fluorine atom, as the (*S*) enantiomer is always obtained (see Scheme 12).

**Scheme 13:**
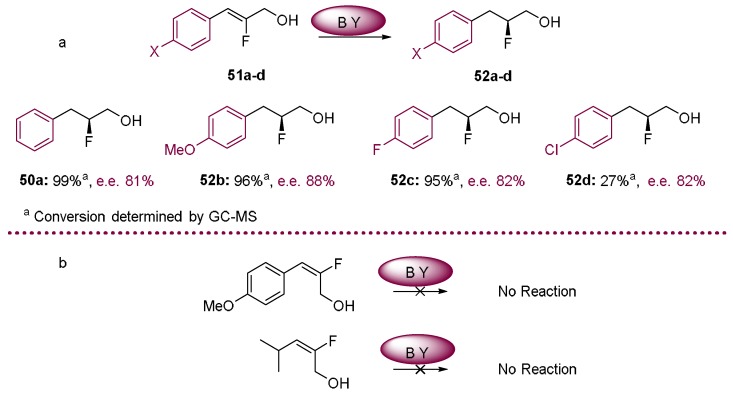
(**a**) Bioreduction of 2-fluoro-2-alken-1-ols; (**b**) failed examples with *E* isomers.

Subsequently, the same authors [37] systematically investigated the scope of substrates for the baker’s yeast-mediated biocatalyzed reduction of 2-fluoro-2-alken-1-ols **51** by switching the (*E*/*Z*) configuration of the alkene moiety and changing the alkyl groups in the β position to the C=C double bond, extending the applicability of this bioreduction reaction. The (*E*/*Z*) configuration of the 2-fluoroallylic alcohol was crucial for the bioreduction yield; thus, the *Z* isomer was almost completely converted to the (*S*) enantiomer within 48 h, with a 91% e.e. (Scheme 13a), whereas the corresponding *E* isomer was not reduced even if the reaction time was prolonged to one week (Scheme 13b). Moreover, alkyl chains with more than seven carbons were less reactive than smaller chains. Generally, other factors, such as the concentration of the substrate, the pH of the medium and the amount of the biocatalyst, also exerted important effects on the reaction rate (see Scheme 13). 

**Scheme 14:**
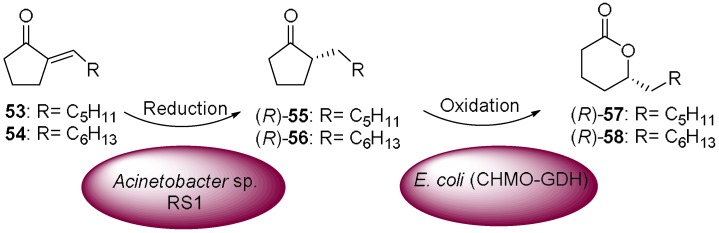
One-pot reduction-oxidation method for the preparation of lactones.

The development of a cascade reaction in one pot has become an advantageous tool in sustainable organic synthesis, mainly because it avoids the requirement for several purification steps, minimizes waste and by-product generation and dramatically decreases the production costs. Several different conditions must be used to perform a specific set of transformations using chemical synthesis. On the other hand, enzymatic processes are more suitable for cascade reactions because of their similar reaction conditions. Consequently, diverse cascade biotransformations have been developed for organic synthesis. In this frame of reference, Li and coworkers [38] reported the first enantioselective reduction-oxidation-hydrolysis cascade for the synthesis of (*R*)-2-alkyl-δ-lactones **57** and **58**, from the corresponding 2-alkylidenecyclopentanones **55** and **56** on 50 mg scale. The authors began by screening microorganisms that reduced compounds **53** and **54** to the cyclic ketones **55** and **56**, respectively, and identified *Acinetobacter* sp. RS1 as an excellent biocatalyst that showed good activity, fast cell growth and high enantioselectivity. Then, in the next step, the authors explored the use of recombinant *Escherichia coli* strains expressing cyclohexanone monooxygenase (CHMO) that had previously been used for Baeyer-Villiger oxidation reactions and identified a reasonable activity for the oxidation of compounds **55** and **56** to the lactones **57** and **58**, respectively. As a result, *Acinetobacter* sp. RS1 and *E. coli* (CHMO) were combined to perform the cascade reduction-oxidation-hydrolysis reaction in one pot. The reaction sequence was conducted by initially reducing the substrates with resting cells of *Acinetobacter* sp. RS1, followed by oxidation with resting cells of *E. coli* (CHMO) to produce compound **57** at a 56% isolation yield and 98% e.e. and compound **58** in a 41% isolation yield and 97% e.e. (see Scheme 14).

Further investigations of asymmetric bioreductions of activated C=C double bonds are ongoing, and future advances will involve the discovery of new and more active biocatalysts, as well as expanding its synthetic utility, providing opportunities to recycle the active microorganisms more efficiently, reducing the reaction time, increasing the reaction scale, etc. These developments will surely expand the applications of the biocatalyzed reduction of C=C carbon bonds. In addition, the reduction of electron-rich double bonds is still a substantial challenge for biocatalysis, and new enzymes and organisms that are able to perform this transformation will certainly be reported in the near future.

## 4. Bioreduction of Prochiral Ketones

Enantiomerically pure secondary alcohols are one of the most relevant precursors for the synthesis of chiral pharmaceuticals, flavors, and agrochemicals, among others. The asymmetric reduction of prochiral ketones is the most forthright method to prepare the desired enantiomerically pure alcohols. Two different approaches for this reduction reaction have been regularly described: the first involves catalytic asymmetric hydrogenation using chiral organometallic complexes, whereas the second implies the use of a biocatalyst, specifically bioreductases. In addition to the well-known chemical methodologies, biocatalytic approaches have gained important, mainly because they embody a more environmentally friendly alternative. In this frame of reference, whole cells from vegetables and microorganisms have been established as efficient biocatalysts for the bioreduction of prochiral ketones. This chapter describes some selected examples using free and supported cells for the enantioselective bioreduction of prochiral ketones with Prelog or anti-Prelog selectivity. We also show a photosynthetic biocatalytic method and a new methodology for bioreductions employing ionic liquids.

**Scheme 15:**
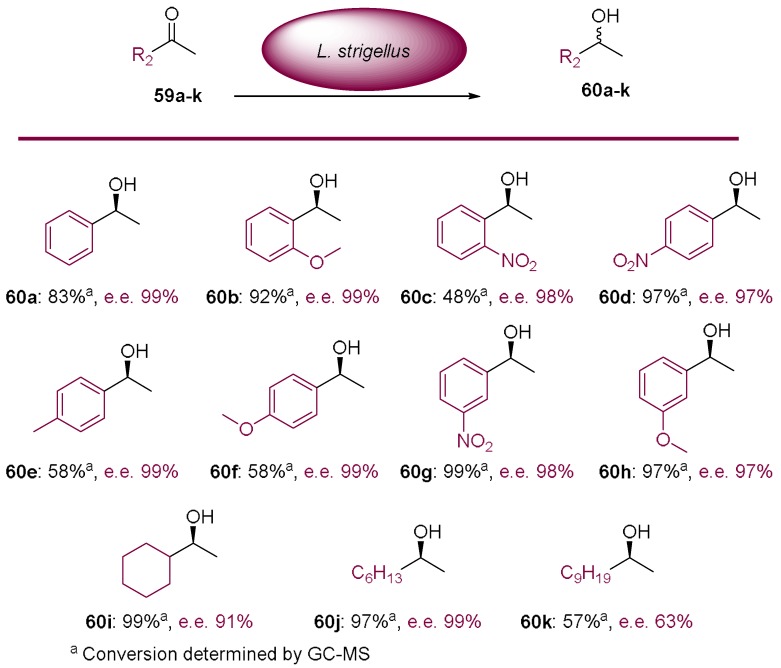
*L strigellus*-biocatalyzed reduction of ketones.

Gotor and coworkers [39] reported an investigation focused on the ability of the basidiomycete *Lentinus strigellus* to serve as a reducing agent of aromatic prochiral ketones (**59**) to the corresponding chiral alcohols (**60**) on 10 mg substrate scale. The authors observed good conversion and excellent enantiomeric excess for almost all the ketones studied. The fungus exhibited Prelog selectivity [40,41] by the insertion of the hydride into the *Re* face of the corresponding ketones. The authors prepared a set of substituted acetophenones and evaluated the influence of electron donor and electron-withdrawing groups in several positions of the aromatic ring. Substituents in the *ortho* position did not significantly affect the enzymatic selectivity, but the electron-withdrawing power of the substituent significantly reduced the reaction yield (compare products **60a** to **60c**), on the other hand, the presence of an electron-withdrawing group in the *para* position of compound **60d** seemed to improve enzymatic activity compared with the reduction of aromatic ketones with electron-donating substituents **60e** and **60f**. Furthermore, the presence of an electron-withdrawing group in the *meta* position of compound **60g** improved the reaction rate. In the case of aliphatic methyl ketones, an increase in the size of the alkyl chain significantly reduced both the conversion and enantiomeric excess of compound **60i** to **60k** (see Scheme 15).

**Scheme 16:**
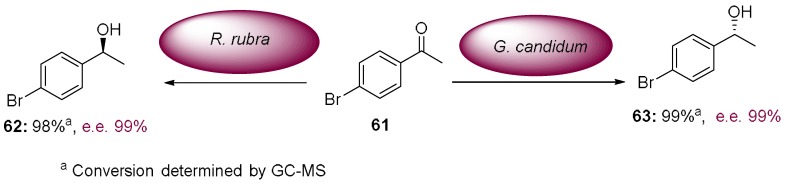
Bioreduction of 4-bromoacetophenone.

Ribeiro et al. published a series of articles examining the use of microorganisms in the asymmetric reduction of different aromatic and aliphatic carbonyl compounds. In the first article [42], the authors evaluated 14 microorganisms (10 yeasts strains and 4 filamentous fungi strains) in the asymmetric reduction of *p*-bromoacetophenone (**61**); all microorganisms were active in the transformation reaction, and the selectivity for the production of (*R*)-enantiomer or (*S*)-enantiomer depended on the microorganisms used in the reduction reaction. The best results were obtained when *Geotrichum candidum* was used. Under the optimized reaction conditions, the conversion rate reached 99% and a 99% e.e. was observed for the (*R*)-isomer (anti-Prelog product), whereas for *Rhodotorula rubra*, the conversion was 98% with a 99% e.e. for the (*S*)-isomer (Prelog product). The scale of the procedure was approximately 50 mg of substrate (see Scheme 16).

**Scheme 17:**
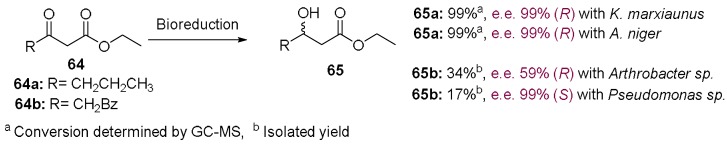
Bioreduction of ethyl 3-oxohexanoate.

In their second paper [43], the authors described the use of seven wild-type microorganism strains for the asymmetric reduction of ethyl 3-oxohexanoate (**64**) to ethyl 3-hydroxyhexanoate (**65**) (an important intermediate in the synthesis of (+)-neopeltolide, a bioactive marine macrolide with potent antiproliferative activity against cancer cell lines). The use of free *Kluyveromyces marxianus* and *Aspergillus niger* cells resulted in conversion rates greater than 99% with 99% e.e. after 24 h; they also observed that after immobilization in calcium alginate spheres, *K. marxianus* cells exhibited essentially the same results, but only after 12 h, and even with relatively high substrate concentrations (10 g/L) on a 500 mg scale, which is potentially very important for industrial biotransformations. This methodology was extended to aryl substituted beta ketoesters by Krotuil and coworkers [44], a screening of several microorganisms led to the identification of some microbial strains that provides access to both enantiomers 3-hydroxy-5-oxo-5-phenylpentanoate (**64b**) with high enantiomeric excess, on a scale of 200 mg of the starting ketone (see Scheme 17).

**Scheme 18:**
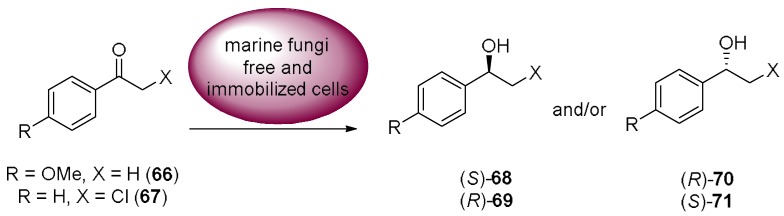
Reduction of ketones by free and immobilized marine fungi.

Porto and his group [45] focused their research on the immobilization and evaluation of biocatalytic processes mediated by whole mycelia of the *P. citrinum* CBMAI 1186 and *Aspergillus sclerotiorum* CBMAI 849 strains using support matrices of silica gel, silica xerogel and chitosan. As mentioned above, the immobilization of the biocatalyst provides several benefits, such as the ability to recover the microorganisms, easy recovery of the product, etc. The free mycelium of *P. citrinum* showed a moderate conversion (40%) and enantioselectivity (69% e.e.) for the reduction of 1-(4-methoxyphenyl)-ethanone (**66**) to the (*R*)-1-(-4-methoxyphenyl)-ethanol (**70**); in stark contrast, the same fungi, *P. citrinum*, immobilized on chitosan catalyzed the reduction of the same substrate with excellent conversion (95%) and enantioselectivity (>99%), affording the corresponding (*S*)-alcohol (**68**) instead of the (*R*)-alcohol. The authors argued that during immobilization, the action of another dehydrogenase may be favored and the bioreduction consequently showed opposite enantioselectivity. On the other hand, whole *A. sclerotiorum* cells immobilized on silica gel and the free mycelium catalyzed the reduction of the ketone **66** to the corresponding (*S*)-alcohol **68** with excellent activity (>99%) and selectivity (>99%). Moreover, whole, free *P. citrinum* cells catalyzed the bioreduction of 2-chloro-1-phenylethanone (**67**) to (*R*)-alcohol (**69**) with poor enantiomeric excess (31%) and good conversion (70%). Additionally, when the reaction was performed using the same microorganism immobilized on silica gel, a stereopreference for the (*S*)-alcohol (**71**) was observed with a similar conversion rate (65%) and enantiomeric excess (25%). The silica xerogel was initially considered as a promissory support for mycelium of filamentous fungi due to its strong adhesion to the mycelia, but actually prevented the access of substrate to the enzyme. The scale used in this study was approximately 75 mg of the corresponding ketone (see Scheme 18).

**Scheme 19:**
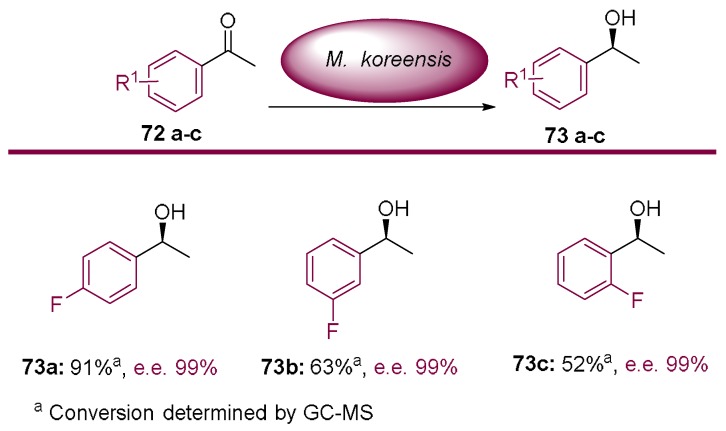
Reduction of acetophenone derivatives by *Metschnikowia koreensis.*

Banerjee and coworkers [46] described the bioreduction of prochiral ketones to enantiomerically pure alcohols catalyzed by freely suspended and supported cells of a recently isolated yeast, *Metschnikowia koreensis* MTCC 5520, using 20 mg of organic substrate. Several immobilization matrices were tested: sodium alginate, calcium alginate, *K*-carrageenan, agarose, polyacrylamide and polyvinyl alcohol. Specifically, the researchers reported that both the freely suspended and gel matrix-entrapped yeast cells were effective biocatalysts for converting prochiral ketones **72** to the corresponding (*S*) alcohols **73**, displaying a Prelog selectivity, with an enantiomeric excess greater than 99%. Using *p*-fluoroacetophenone (**72a**), the conversion was almost complete at 25 °C within 3 h at pH 9, and the supplementation of the reaction mixture with 20 g/L glucose markedly improved the rate of the bioreduction reaction, probably by accelerating the cofactor recycling process in the cells. The microorganisms were able to reduce various acetophenones substituted with electron-withdrawing groups on the phenyl ring, showing a significant activity when an EWG was located at the *para* position compared to *ortho* and *meta* positions, consistent with previously reported results and representing a general phenomenon in the reduction of substituted acetophenones. Additionally, the thermal stability and the substrate tolerance of the yeast were improved by immobilization in calcium alginate beads (see Scheme 19).

**Scheme 20:**
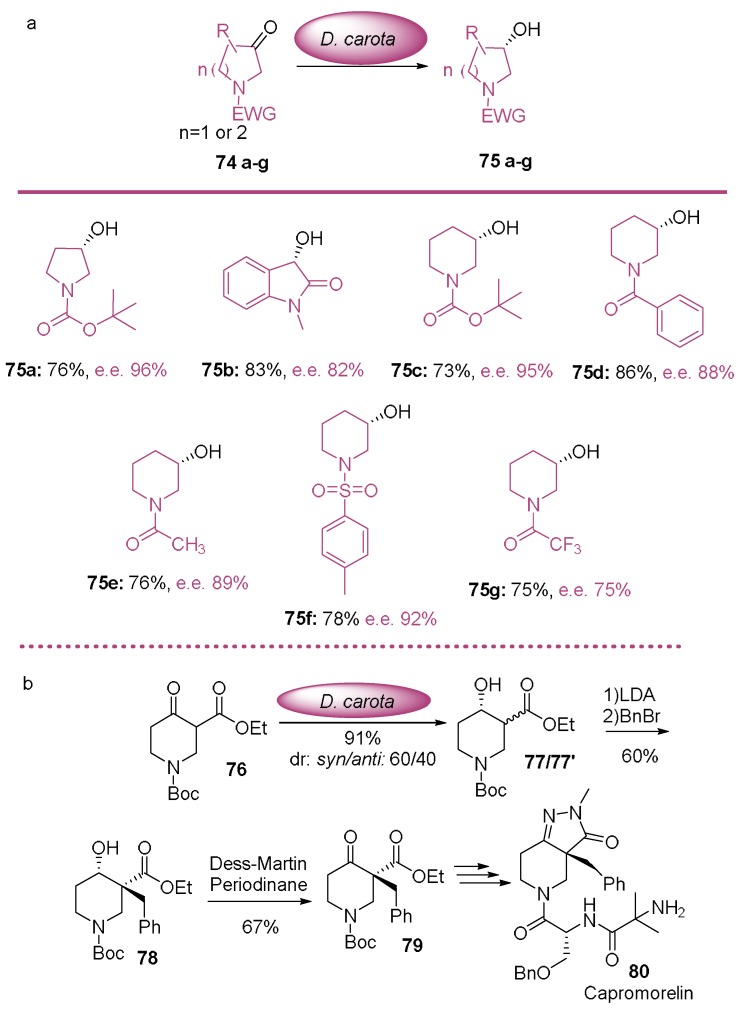
(**a**) Reduction of cyclic amino ketones by *D. carota*; (**b**) Synthesis of capromorelin precursor.

At this point, whole cells are able to perform bioreductions of carbonyl compounds and that their use is an interesting alternative to the classic chemical processes. Furthermore, these biocatalysts have a ubiquitous distribution and are easily accessible. Unfortunately, these bioreductions are commonly accomplished using monofunctional substrates and, in most cases, very simple starting materials. As we mentioned above, the main reason is that the enzymatic machinery of whole cells can catalyze unwanted reactions. A very interesting paper published by Cossy and coworkers [47] reported the use of carrots (*Daucus carota*) to reduce cyclic amino-ketones **74**; to the best of our knowledge, that paper was the first example of the bioreduction of amino ketones and the first to use of protecting groups. The authors showed high yields and high enantiomeric excesses, obtaining the corresponding 3-hydroxypyrrolidines **75a**,**b** and 3-hydroxypiperidines **75c**–**h** with the (*S*) configuration of the stereogenic center at C3 on a typical scale of 450 mg of organic substrate; these results were consistent with the Prelog rule (see Scheme 20a). Then, the same biocatalytic reduction reaction was applied to the synthesis of an advanced intermediate in the production of an antiaging drug known as capromorelin (**80**); the authors performed the transformation of compound **76** to compounds **77**/**77′**) using *Daucus carota* (yield: 90%, d.r.: 60:40, e.e.: 91% for each individual diastereoisomers). The mixture of compounds **77**/**77′** were diastereoselectively alkylated by benzylbromide using LDA to produce compound **78** in 60% yield; this compound was oxidized using Dess-Martin periodinane to yield compound **79**, which is a known precursor of compound **80** (see Scheme 20b).

Currently, most of the whole cell biocatalytic processes used for the reduction of prochiral ketones follow Prelog’s rule. Consequently, the (*S*) alcohols are usually obtained when the smaller substituent of the ketone has a lower Cahn-Ingold-Prelog priority; thus, the preparation of (*R*) alcohols, which are as important as their (*S*) enantiomers, is still of tremendous interest in biocatalysis. Therefore, Luo and his team [48] conducted a successful biocatalytic anti-Prelog reduction of 2-octanone (**81a**) to (*R*)-2-octanol with high enantioselectivity using whole *Acetobacter pasteurianus* GIM1.158 cells. Ten additional prochiral compounds **81b**–**k** were selected to rationally evaluate the potential of this microorganism to reduce carbonyl compounds. *A. pasteurianus* was able to catalyze the anti-Prelog asymmetric reductions of all tested ketones to the corresponding alcohols **82a**–**k** with more than 92% e.e.

**Scheme 21:**
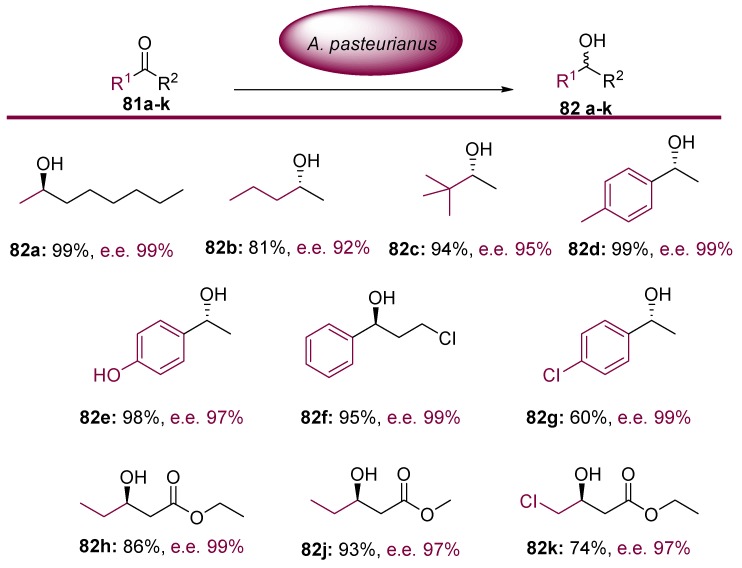
Reduction of ketones by *A. pasteurianus*.

Using aliphatic ketones, the yield and the enantiomeric excess increased for substrates with longer chains. In the case of prochiral keto esters, the biocatalyst also showed high catalytic activity and enantioselectivity; likewise, a 200 mL preparative scale bioreduction of 1 g of 2-octanone (**81a**) to (*R*)-2-octanol was performed, obtaining a 95% yield and greater than 99% e.e. in 70 min. Those results were much better than previous biotransformations reported for the same reaction (see Scheme 21).

**Scheme 22:**
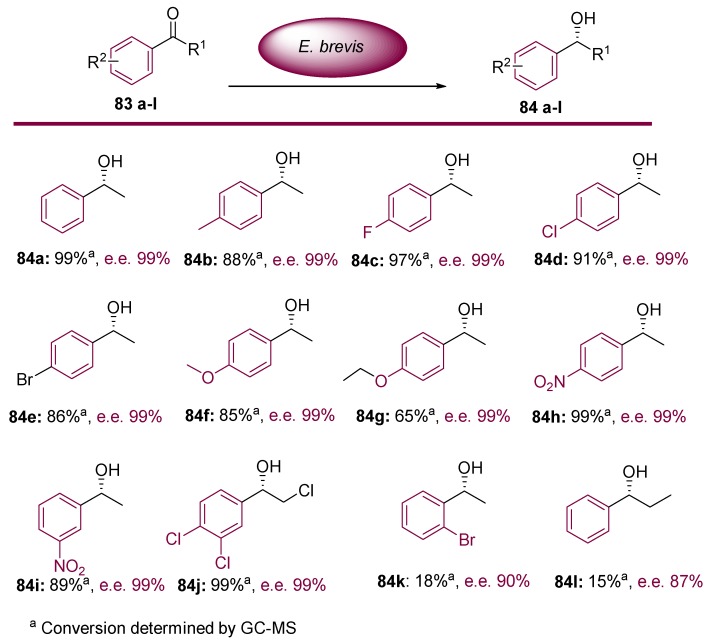
Reduction of ketones by *E. brevis*.

In a similar study exploring biocatalysts with anti-Prelog selectivity, Yu and coworkers [49] described the activity and enantioselectivity of the reduction of prochiral ketones **83** mediated by a recently isolated bacterial strain, *Empedobacter brevis* ZJUY-1401. The microorganism was highly active for the reduction of acetophenones, producing the corresponding alcohols **84** with excellent enantiomeric purity (>99%). The activity of the biocatalyst was clearly influenced by the substituents present in the aromatic ring of the acetophenone, as some substituents, such as methyl, halogen and alkoxy groups, decreased the activity and afforded lower conversion rates. Substrates with either electron-withdrawing or electron-donating groups in the *para* position produced excellent enantioselectivities; however, all of the substrates exhibited lower conversion rates than that of the unsubstituted acetophenone. The authors postulated that steric hindrance was responsible for the low conversion rates. Additionally, the activity of the process was influenced by the position of the substituents on the benzene ring; as a general trend, rings with substitutions in the *ortho* or *meta* positions needed longer reactions times and afforded lower yields than their *para*-substituted counterparts. These experiments were conducted on a scale of 20 mg of the corresponding ketone (see Scheme 22).

**Scheme 23:**
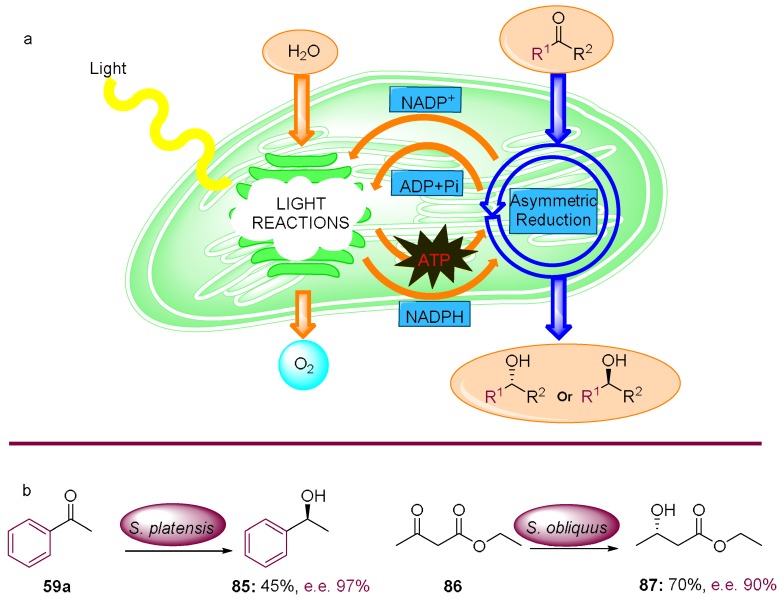
(**a**) Photosynthetic biocatalytic process; (**b**) Reduction of ketones by microalgae-mediated photocatalysis.

Exploring new alternatives to regenerate the cofactors in biocatalytic reactions, the Wang group [50] studied the use of microalgae in the photobiocatalytic asymmetric reduction of prochiral ketones. Photosynthetic organisms are able to capture light energy to produce NADPH from NADP^+^ through photosynthetic electron transfer reactions. A parallel process is the synthesis of sugar from CO_2_, generally using NADPH. The reducing power of NADPH produced during photosynthesis has also been used to reduce exogenous substrates. The authors chose ethyl acetoacetate (**86**) and acetophenone (**59a**) as model substrates for β-ketoesters and aromatic ketones, respectively. The substrates were reduced to the corresponding (*S*) alcohols by prokaryotic cyanophyta and eukaryotic chlorophyta in photobiocatalytic processes with high enantioselectivity. For aromatic ketones, whole *Streptomyces platensis* cells were a suitable biocatalyst, as a 45% yield and 97% e.e. were achieved. On the other hand, a 70% yield and 90 e.e. were attained when *Scenedesmus obliquus* was used in reactions with β-ketoesters. Both bioreduction reactions were remarkably improved by the addition of glucose as a cosubstrate, providing evidence for the ability of those microorganisms to use carbohydrates to regenerate the cofactor NAD(P)H by respiratory metabolism. The scale used was approximately 150 mg of the carbonyl compound (see Scheme 23).

**Scheme 24:**
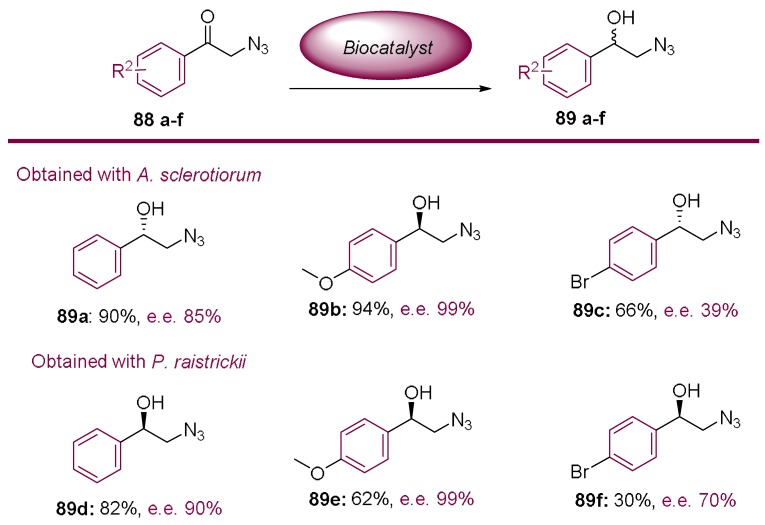
Reduction of α-keto azides by marine microorganisms.

Marine organisms such as mollusks, seaweeds, sponges and microorganisms express sets of enzymes that display very useful characteristics, such as stability at different pH values and temperatures, making them suitable biocatalysts. Based on these findings, Porto et al. [51] explored the use of several strains of marine-derived fungi as biocatalysts for the asymmetric reduction of α-keto azides **88** to their corresponding β-azidophenylethanols **89**, which are important precursors of chiral aziridines and amino alcohols used in drug synthesis. The marine fungi showed Prelog and anti-Prelog selectivities with good conversions and excellent enantiomeric excess. For example, the fungus *Aspergillus sclerotiorum* CBMAI 849 was able to catalyze the reduction of several α-azido ketones and produced different enantiomers, depending on the substrate. Similarly, the fungi *Penicillium raistrickii* CBMAI 931 catalyzed the reduction of several α-azido ketones and produced different enantiomers, depending on the substrate. For this study, 100 mg of organic substrate was used (see Scheme 24).

The extensive use of conventional organic solvents in biocatalytic processes may frequently suffer from limitations such as toxicity to the biocatalyst and the environment; in this scenario, ionic liquids (ILs) have recently appeared as a new class of relatively biocompatible solvents. In contrast to traditional organic solvents, ILs are nonflammable, nonvolatile, highly stable, and are able to dissolve nonpolar and polar compounds. An additional benefit of this kind of solvents is that their properties can be altered to fit the specific requirements of a process by structuring the anions and cations. Consequently, the use of ILs in biocatalytic processes has recently been extensively studied [52,53], showing excellent results in terms of activity, enantioselectivity and stability of the biocatalyst. Lou and coworkers [54] synthesized enantiopure alcohols **91** via the anti-Prelog asymmetric reduction of ketones **90** with whole *Acetobacter* sp. CCTCC M209061 cells, improving the process using hydrophilic ionic liquids and typically approximately 500 mg of the corresponding ketone. 

**Scheme 25:**
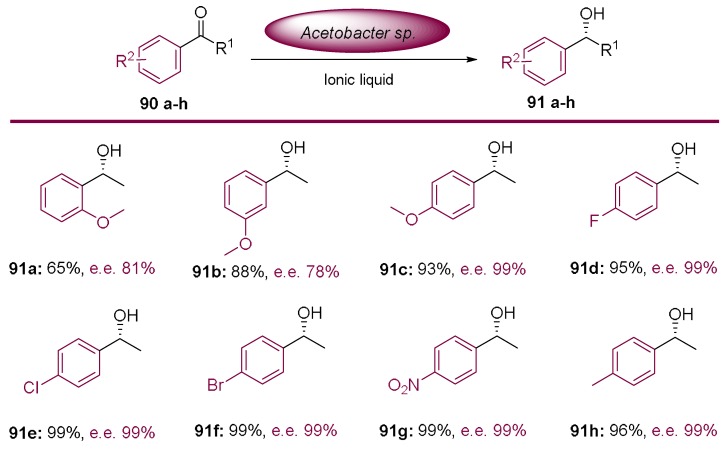
Reduction of ketones by *Acetobacter* sp. in ionic liquids.

The best results were obtained with 1-(2′-hydroxyl) ethyl-3-methylimidazolium nitrate (C_2_OHMIM.NO_3_), which showed good biocompatibility and increased the cell membrane permeability. The authors reported a maximum yield of 91% and an e.e. greater than 99%. Several aryl ketones with different substituents were tested, and the cells were able to catalyze the anti-Prelog enantioselective reductions of all tested ketones to the corresponding (*R*)-alcohols with excellent enantiomeric excess in most cases. Moreover, the substituents attached to the aromatic ring had a significant impact on the bioreduction reaction. The electron-withdrawing substituents (-NO_2_ and halogens) substantially increased the initial reaction rate, probably by lowering the electron cloud density surrounding the aromatic ring and consequently contributing to the nucleophilic reduction. The position of the substituents also affects the bioreduction, as the rate of the process is sensitive to steric hindrance in the *ortho* position (see Scheme 25).

The use of biocatalysis as an alternative option for the enantioselective synthesis of chiral alcohols is currently of undeniable significance, and some recent developments have adopted this strategy as the primary option for the construction of fine chemicals or advanced intermediates in organic chemistry. However, as it is common in biocatalysis, many opportunities exist to explore and improve the future applications of this methodology.

## 5. Oxidation of Sulfides

Previous chapters of this review have discussed asymmetric enzymatic reduction reactions performed with whole cells; however, oxidation reactions, although they are less common, are also important chemical transformations that have been conducted using enzymes and whole cells. In recent years, enantioselective oxidation of prochiral sulfides performed by biocatalyst has attained considerable interest because chiral sulfoxides have a great variety of applications in chemistry and pharmaceutical industries. Optically pure sulfoxides are usually prepared by chemical methods, among which transition metal catalysis and organocatalysis are commonly employed. Nevertheless, the application of pure isolated enzymes is also effective; these three strategies are limited by cost and difficulties related to the isolation and long-term stability of enzymes, respectively. In contrast, asymmetric sulfoxidation reactions catalyzed by whole-cell systems are much cheaper and avoid the use of expensive cofactors. This section describes several examples of the enantioselective oxidation of sulfides to the corresponding sulfoxides using different microorganisms as the biocatalyst, as well as the use of biphasic systems to increase the efficiency of this kind of oxidations. Additionally, some potential pharmaceutical applications of this methodology are described.

**Scheme 26:**
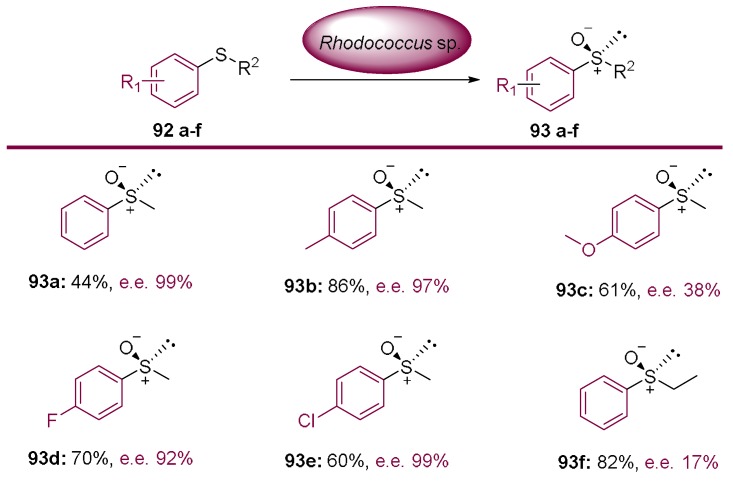
Oxidation of sulfides by *Rhodococcus* sp.

Xu and coworkers [55] reported the catalytic performance of a newly isolated bacterium, *Rhodococcus* sp. strain ECU0066, in the asymmetric oxidation of aryl sulfides **92** to the corresponding sulfoxides (**93**). This type of biotransformation reaction is not particularly commonly performed using bacteria, as fungi have been frequently used for this purpose; however, the authors indicated that the biocatalyst displayed a fairly high activity and stereoselectivity for most of the sulfides tested. The oxidation of simple monosubstituted aromatic sulfides (using approximately 50 mg of substrate) was performed with an excellent e.e. (99%) and a moderate yield (44%) of compound **93a**, with only traces of the corresponding sulfone. Notably, in chemical oxidation reactions, the production of sulfones is a common issue that is difficult to avoid. In the case of *para*-substituted substrates, high yields and almost enantiopure (*S_S_*) sulfoxides were obtained; the authors also proved that the enantioselectivity of the process does not depend on the electronic properties of the *para* substituents, since excellent enantiomeric excess was obtained with electron-withdrawing and electron-donating groups. On the other hand, the stereoselectivity of the bioreduction reaction was dramatically decreased as the size of the alkyl side chain increased, see compounds **93a** and **93f** (Scheme 26).

**Scheme 27:**
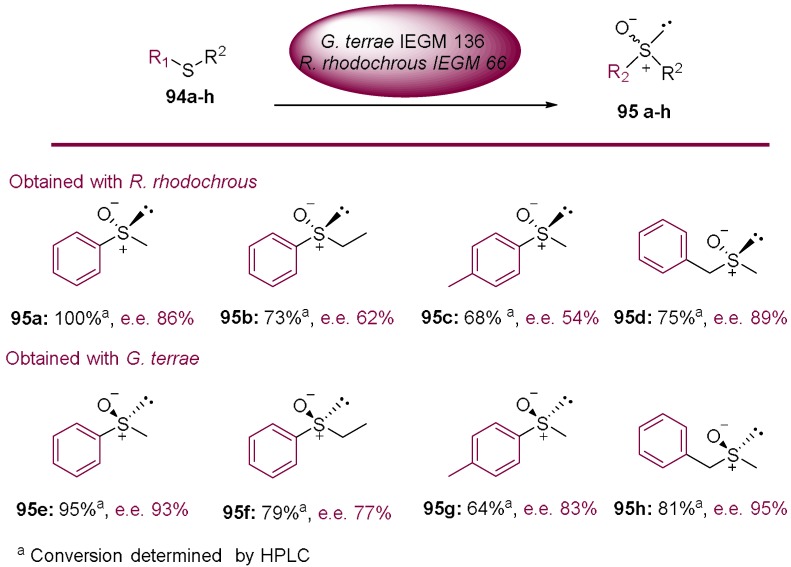
Oxidation of sulfides by *R.* *rhodochrous* and *G. terrae.*

Later, Elkin and his group [56] optimized the conditions for the oxidation of methyl phenyl sulfide (**94**) by the application of the bacteria *Rhodococcus rhodochrous* IEGM 66 and *Gordonia terrae* IEGM 136, studying the effects of pH of the culture medium, sulfide concentration, temperature and aeration regimes. In a 50 mg scale reaction, the selected biocatalysts were highly active toward methyl phenyl sulfide and its analogs ethyl phenyl, methyl *p*-tolyl, and benzyl methyl sulfides, reaching yields between 64–100% and enantiomeric excess ranging from 54% to 95%. Interestingly, *R. rhodochrous* IEGM 66 enabled the production of alkyl aryl sulfoxides (**95**) with a (*S_S_*)-asymmetric center, while *G. terrae* lead to the formation of alkyl sulfoxides with a (*R_S_*)-asymmetric center (see Scheme 27).

**Scheme 28:**
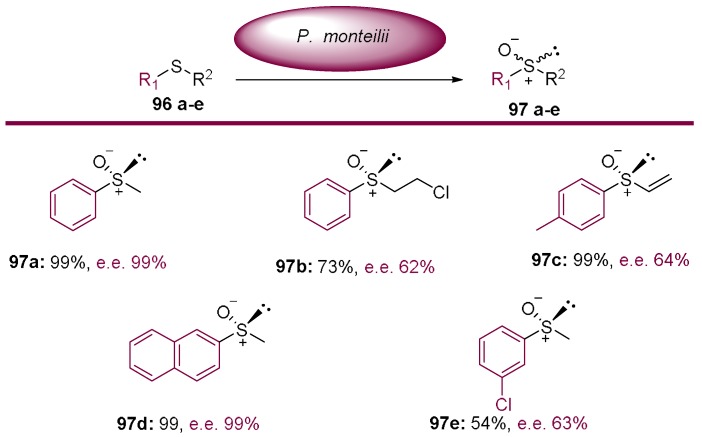
Oxidation of sulfides by *P. monteilii.*

Biphasic systems with whole-cell biocatalysts are suitable for the partitioning of the substrate and the product between the two phases and enable the use of higher concentrations and avoid substrate/product inhibition, thus reducing their toxic effects on the living biocatalysts and improving the overall effectiveness of the process. Based on this principle, Chen et al. [57] investigated the ability of *Pseudomonas monteilii* CCTCC M2013683 to perform the selective oxidation of aryl sulfides **96** and obtained excellent activity, stereoselectivity and high substrate tolerance using an *n*-hexane-aqueous buffer (1:1) as the biphasic system for the transformation reaction. The optimized reaction conditions, such as substrate concentration, cell density, reaction temperature, pH medium and co-solvent system, were established and applied to the biocatalytic oxidation of a broad series of sulfides on an 80–100 mg scale. The yields of the chiral sulfoxides **97** ranged between 54% and 99% in 63–99% enantiomeric excess for the corresponding (*R_S_*) enantiomer, and no undesired over-oxidation to sulfones was observed (see Scheme 28).

**Scheme 29:**
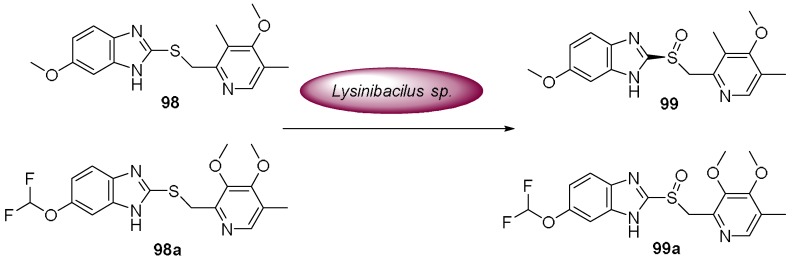
Biooxidation of esomeprazole sulfide and related compounds.

One of the most relevant pharmaceutical applications of the whole-cell oxidation of sulfides is the production of esomeprazole (**99**) (*S*-enantiomer) from the sulfide **98**; this compound is an API used to treat gastroesophageal reflux disease and gastric ulcers. Although several fungi and bacteria have been used as catalysts to produce the (*R_S_*) enantiomer of omeprazole, a microorganism capable of performing the biocatalytic synthesis of compound **99** has not been reported. Furthermore, since the synthesis of enantiopure (*S_S_*) sulfoxide is laborious even when using the improved chemical methods, its microbial preparation has received considerable attention over the past years as a cost-effective alternative. In this context, Stepanek and coworkers [58] described a screen for new microbial strains that are able to perform the enantiopure oxidation of the corresponding prochiral sulfide to compound **99** and identified a bacterial strain belonging to the genus *Lysinibacillus* that was able to catalyze the enantioselective oxidation with a 77% yield and 99% e.e. on a 200 mg scale without the subsequent formation of the undesired sulfone. The researchers tested more than one thousand bacterial strains, and 22 strains were able to transform compound **98** into the (*R_S_*) enantiomer and only five catalyzed the production of compound **99**. Additionally, using the same biocatalyst, the authors performed the oxidation of the closely related substrate **98a**, which is the sulfide precursor of the active pharmaceutical ingredient pantoprazole **99a**, indicating that this processes is highly specific, as minimum modifications in the substrates lead to dramatic changes in the catalytic activity (see Scheme 29).

**Scheme 30:**
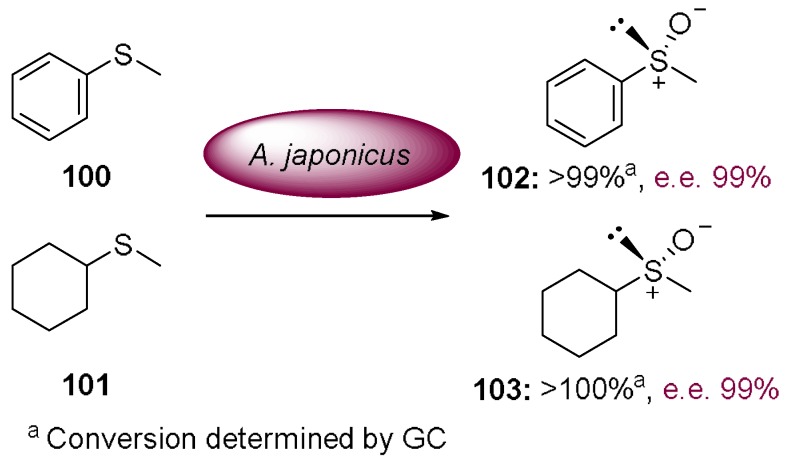
Oxidation of sulfides by *A. japonicus.*

Kurina-Sanz and coworkers [59] expanded our knowledge of the ability of filamentous fungi to selectively convert sulfur-containing compounds, revealing that several species of the *Aspergillus* genus are able to perform chemo- and stereoselective oxidation of organic sulfides. The authors used two different substrates as models: aromatic thioanisole **100** and aliphatic cyclohexyl(methyl)sulfide (**101**). All nine tested strains effectively oxidized the aliphatic sulfide, and most of them showed the ability to oxidize the thioanisole. However, the aromatic sulfide proved to be a problematic substrate. For example, the e.e. values for the corresponding aromatic sulfoxide did not remain constant when *Aspergillus flavus*, *Aspergillus niger* and *Aspergillus fumigatus* were employed. In all cases, the (*R_S_*)-enantiomer was detected exclusively, and full chemoselectivity was verified since no sulfone was detected. Among the studied strains, *Aspergillus japonicus* ICFC 744/11 was an auspicious whole-cell biocatalyst for the preparation of enantiopure sulfoxides, since after determining the optimal conditions using a substrate concentration of 0.1 mM, both the conversion (100%) and optical purity (99% e.e.) of (*Rs*)-cyclohexyl(methyl)sulfoxide were exceptional (see Scheme 30).

The application of biocatalysts to the chiral oxidation of sulfides is a rapidly growing area of the wide spectra of biologically mediated chemical transformations because their implementation often results in high yields and high levels of enantioselectivity and chemoselectivity. Further studies aiming to discover new biocatalysts and substrate scopes and optimize reaction parameters are surely the focus of research in several laboratories around the world.

## 6. Other Transformations

In this section, we discuss some additional examples of the use of whole cells for chemo-, stereo- and/or enantioselective transformations performed by both naturally occurring and engineered microorganisms, with the aim of showing the ever-expanding range of products that can be obtained using these methodologies. Additionally, some recent developments in the biocatalytic preparation of several substances with potential industrial and synthetic applications are described.

**Scheme 31:**
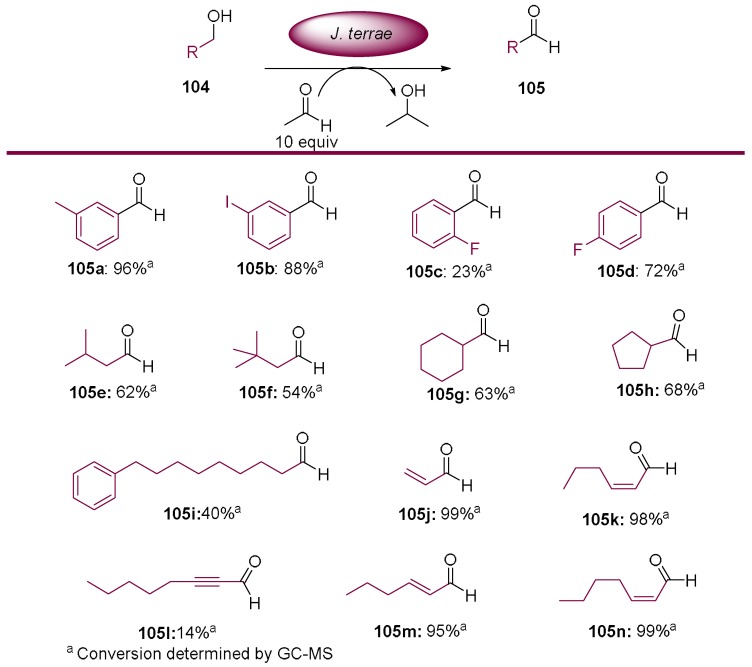
Oxidation of alcohols by *J. terrae.*

One of the basic reactions in synthetic organic chemistry is the selective oxidation of alcohols to yield carbonyl compounds. Recently, a strong emphasis has been placed on developing greener alternatives to the typical chemical procedures. Therefore, Krotuil and his group [60] reported a study on the substrate spectrum of the lyophilized cells of *Janibacter terrae* DSM 13953 for the chemoselective oxidation of alcohols to aldehydes **105** in a hydrogen-transfer process. In this reaction, the *Janibacter terrae* cells work as a biocatalyst for the hydrogen transfer process using acetaldehyde as a hydride acceptor. Benzyl alcohols with substituents in the *meta* position were better substrates than those with substituents in the *ortho* or *para* positions. Moreover, the size of the substituents in the *ortho* position in the benzyl alcohols is important, since these substrates were not transformed, except for substrates with small substituents such as F, Me and OH. The size of the substituent in the *para* position also exerts a relevant effect on the conversion; again, substrates with small substituents (F and Me) are transformed faster than substrates with large substituents (I and Ph). The researchers did not observe a clear electronic effect on the biotransformation reaction. Similarly, disubstituted benzyl alcohols and their heteroaromatic analogs are generally less reactive; furthermore, a significant preference for primary alcohols was observed when the process was compared with the oxidation rate of secondary alcohols. Switching to aliphatic alcohols, the branched alcohols were less reactive than linear substrates; likewise, allylic alcohols were generally excellent substrates. The transformation reactions conducted in this study were performed on a 50–100 mg scale (see Scheme 31).

**Scheme 32:**
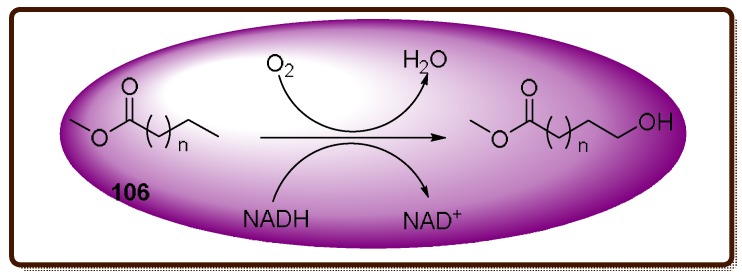
Terminal and unactivated C-H bond oxyfunctionalization in FAMEs by whole cell biocatalysis.

Another important related application of biocatalysis is the possibility that this methodology can perform regiospecific oxidation of unactivated substrates. Recently, a biocatalytic procedure was evaluated as a method to expand the synthetic utility of fatty acid methyl esters (FAMEs) **106**, which constitute a renewable feedstock for the chemical industry, but at the same time are poorly exploited in synthesis reactions because of their inactive character, such as the inert character of the sp^3^ C-H bond. Bülher and coworkers [61] studied the oxyfunctionalization of FAMEs using recombinant *E. coli* as a biocatalyst. The bioengineered catalytic microorganism contained a plasmid from *Pseudomonas putida* that encodes all the genes of the alkane degradation pathway. FAMEs ranging from pentanoic acid methyl ester to dodecanoic acid methyl ester were converted by the biocatalyst. This approach catalyzed the hydroxylation reaction exclusively at the ω-position, leading to the formation of terminal alcohols **107**, and in a few cases, the production of relevant concentrations of the corresponding aldehydes. The best result was obtained when nonanoic and decanoic acid methyl ester were used as substrates on a 0.5 mg scale (100% conversion determined by GC-MS), with a 95% yield to the corresponding alcohol and elevated conversion rate, representing a good starting point for the preparative synthesis of terminally functionalized FAMEs in a biotransformation process (see Scheme 32).

**Scheme 33:**
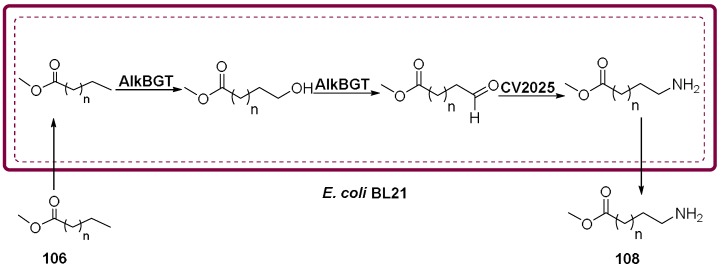
Terminal amino-functionalization of dodecanoic acid methyl ester with *E. coli* Bl21.

This regiospecific functionalization of unactivated substrates was subsequently expanded to the amination of non-activated C-H bonds of FAMEs **106** and some alkanes. Using one recombinant *E. coli* catalyst [62], the researchers coupled oxygenase and transaminase catalysis in vivo, finding that both substrates were converted to the corresponding terminal amines **108** in a reaction performed on a 0.5 mg scale, with absolute regiospecificity via two sequential oxidation reactions and one amination step. The synthesis of the aminated compounds occurred through a consecutive three-step reaction in one recombinant microbe expressing the genes for alkane monooxygenase AlkBGT from *Pseudomonas putida* and ω-trans aminase CV2025 from *Chromobacterium violaceum*, but the conversion rates were not particularly high (5% for FAMEs and 15% for alkanes). However, this example constitutes proof of the versatility of microbial catalysis for the specific functionalization of non-functionalized carbons, which is a difficult process to achieve using chemical methods (see Scheme 33).

**Scheme 34:**
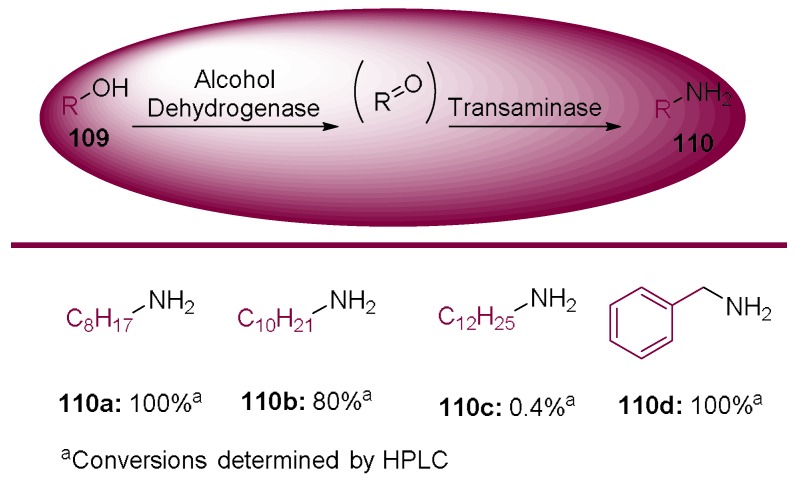
Biocatalyzed amination of alcohols.

Alternatively, Wendish et al. [63] exploited the inherent high reactivity of the biosynthesized aldehydes through the production of non-natural mono and diamines **110** using a designed *E. coli* whole cell as a biocatalyst. A three-enzyme cascade employing alcohol dehydrogenase, ω transaminase and l-alanine dehydrogenase was used for redox self-sufficient amination of alcohols **109**. In the first step, 0.1 mg of the alcoholic substrate is oxidized to the intermediate aldehyde, which is immediately aminated by an l-alanine-dependent transaminase; the versatility of this cascade approach was evaluated using several primary and secondary linear alcohols and diols, as well as cyclic and aromatic alcohols. The best results were obtained using 6-hexanol, 1,10-decanodiol and benzyl alcohol, resulting 100% conversion. However, other alcohols proved to be good substrates, producing the corresponding mono or diamine. Moreover, the selectivity for the transformation of primary alcohols was very high, since very low concentrations of secondary amines were observed, even after longer reaction times (see Scheme 34).

**Scheme 35:**
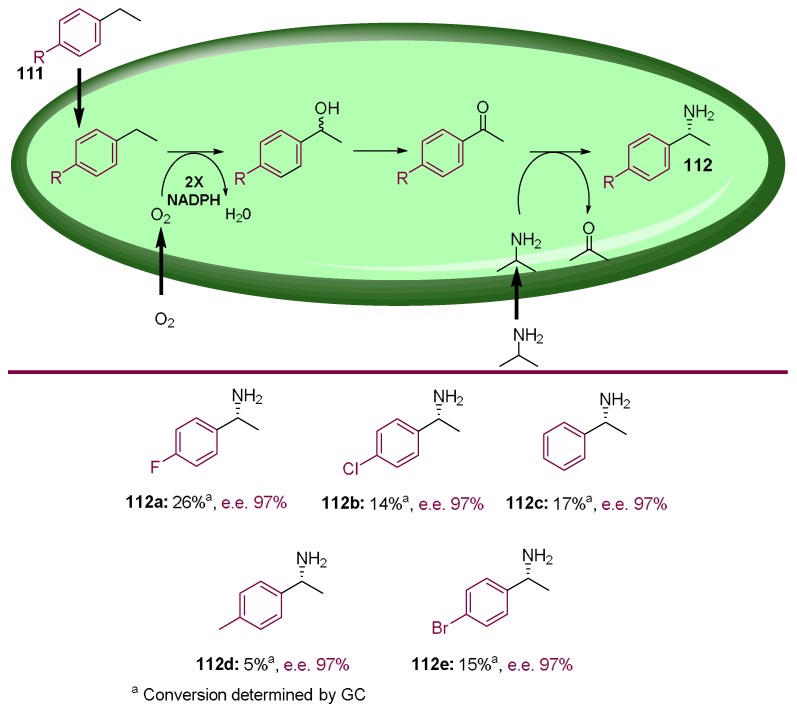
Conversion of 4-substituted ethylbenzenes into amines catalyzed by *E. coli* Bl21.

Afterwards, an even more ambitious approach for the application of a multi-enzyme cascade to the stereoselective amination of unactivated benzylic alkenes **111** was developed by Both and coworkers [64]. The researchers engineered a single *E. coli* bacterial whole cell as a biocatalyst. Importantly, the most successful chemical strategies used to complete this transformation frequently involve transition-metal-catalyzed reactions. In contrast, an initial C-H activation by a self-sufficient cytochrome P450 monooxygenase was envisaged to produce the intermediated benzylic alcohol; then, this substrate was oxidized to the corresponding ketone using two different alcohol dehydrogenases with complementary stereoselectivity. This step was necessary since C-H activation is not always stereoselective. Finally, a transaminase was used to obtain the amine with the desired stereoselectivity; this process represents the first example of a four-enzyme cascade. The results showed the conversion of typically less than 0.1 mg of starting material **111** to enantiomerically pure (*R*)-phenylethanamines **112** with a conversion rate of up to 26%. Under these reaction conditions, no additional cofactor, except for the amine donor isopropyl amine and molecular oxygen, was required (see Scheme 35).

**Scheme 36:**
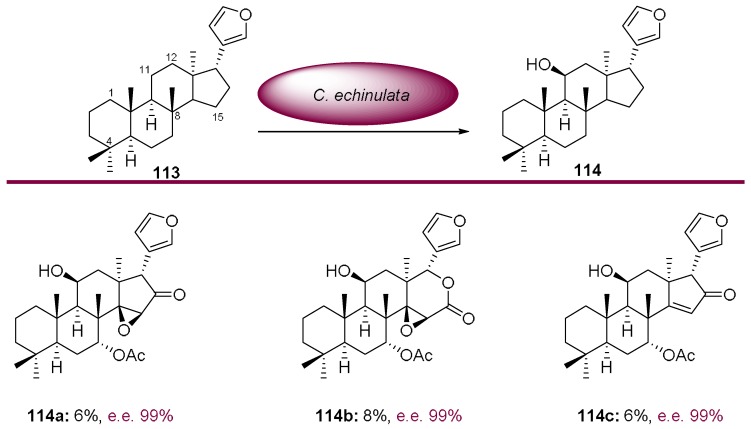
Hydroxylation of the basic limonoid skeleton by *C. echinulata.*

As an example of the application of biocatalysis in the semi synthesis of natural products, particularly when reactions must be performed on unactivated positions of a particular substrate, Thulasiram and coworkers [65] explored the regio- and stereoselective 11β-hydroxylation on the basic limonoid skeletons **113** using whole cells of *Cunninghamella echinulata* as biocatalyst and 700 mg of starting material. Notably, the steroidal carbon skeletons of limonoids are rich in chemically sensitive oxygenated functionalities and display high skeletal complexity; consequently, high efficiency and chemo-, regio- and stereoselectivity are required for the desired transformation. The aforementioned microorganism converted limonoids to their 11 β-hydroxy analogs **114** as the sole products, with yields of approximately 10%. Moreover, the position of the hydroxylation corresponds to an inactivated carbon; as a result, this reaction is at best time consuming when performed using traditional chemical methodologies (see Scheme 36).

One of the best applications of biocatalysis is the preparation of enantiopure vicinal diols, compounds that are useful and valuable synthetic intermediates for many bioactive compounds, pharmaceuticals and chiral reagents. Asymmetric *cis*-dihydroxylation of olefins can be achieved in one step by Sharpless dihydroxylation using a heavy metal oxide catalyst with chiral ligands; on the other hand, chemical *trans*-dihydroxylation of olefins requires two reaction steps: epoxidation and the subsequent epoxide hydrolysis. This strategy has been used to purify unstable intermediates and uses toxic metals (Os or Co, among others). Thus, one-pot cascade biocatalysis for this transformation offers a greener and perhaps more efficient approach for the preparation of vicinal *trans*-diols. The approach is also a complementary tool to Sharpless dihydroxylation. Using this strategy, Li and coworkers [66] reported the development of *E. coli* (SSP1) cells coexpressing a styrene monooxygenase and an epoxide hydrolase as a simple and efficient biocatalyst for the (*S*)-enantioselective dihydroxylation of several terminal aryl olefins **115** to produce the corresponding (*S*)-vicinal diols **116** with a high e.e. and good yield. Additionally, the development of *E. coli* (SST1) cells co-expressing the same styrene monooxygenase and another epoxide hydrolase with the complementary regioselectivity has been reported as an efficient and simple biocatalyst for the preparation of the corresponding (*R*)-vicinal diols **117** with good yield and a high e.e.

**Scheme 37:**
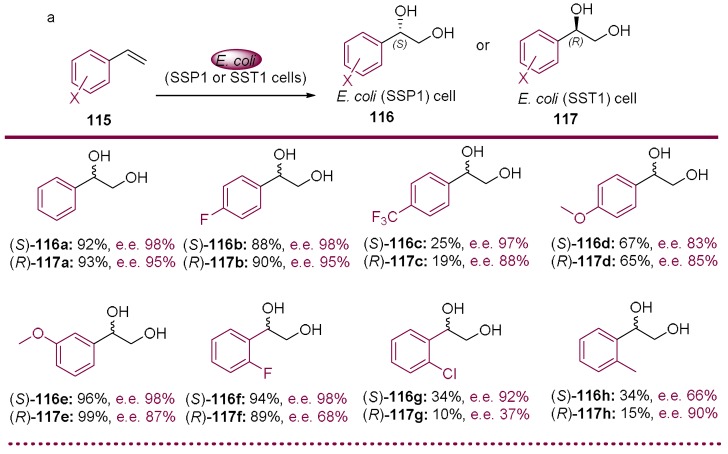

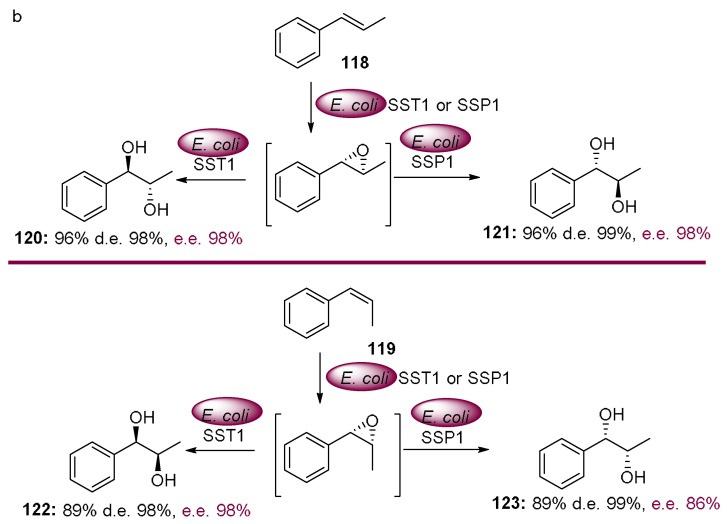
(**a**) Biocatalyzed enantioselective dihydroxylation of aryl olefins; (**b**) Biocatalyzed epoxidation-hydration sequence.

The authors used resting cells of both *E. coli* strains for the dihydroxylation of styrene and related substrates (approximately 20 mg) in a two-phase liquid system containing phosphate buffer and *n*-hexadecane. The high enantioselectivity of dihydroxylation processes is attributed to the high selectivity of styrene monooxygenase and high regioselectivity of the hydrolases in the hydrolysis of the corresponding epoxides at the β-position. Some problematic substrates were characterized by the presence of strong electron-withdrawing groups or an *ortho* substituent, and the authors argued that these characteristics decreased the activity of the first enzyme, producing a low epoxidation activity (Scheme 37a). 

**Scheme 38:**
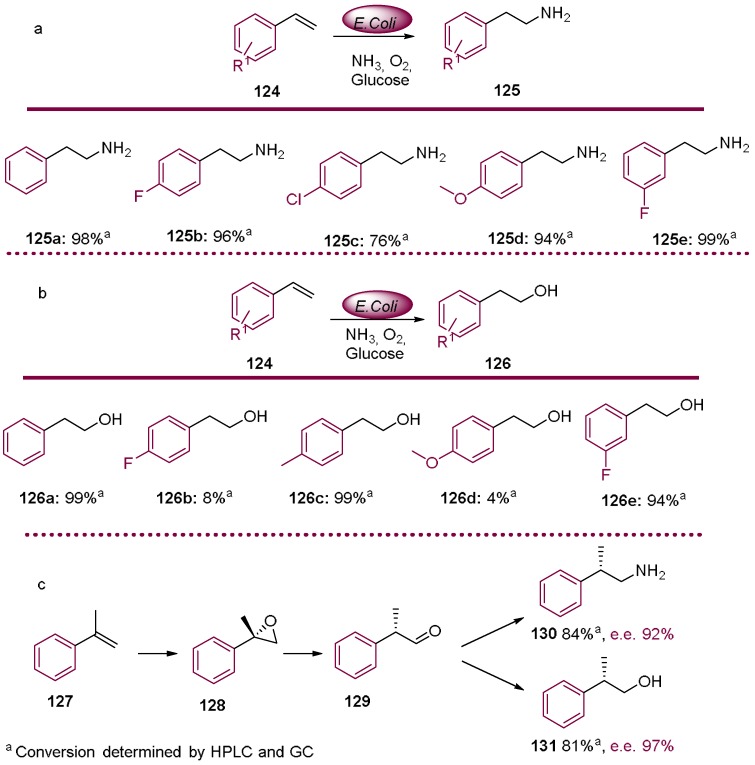
(**a**) Synthesis of terminal amines; (**b**) anti-Markovnikov hydration of aryl olefins; (**c**) Enantioselective hydroamination and hydration of aryl olefins.

The researchers performed the *trans*-oriented transformation of the non-terminal olefins **118** and **119** to produce **120** and **122** in excellent enantiomeric and diastereomeric excess (>98% each) using *E. coli* (SST1) as biocatalyst to prepare vicinal diols with two chiral centers. On the other hand, *trans*-hydroxylation of **118** and **119** with *E. coli* (SSP1) afforded the products **121** and **123** with very high enantiomeric and diastereomeric excess as well. The greatest accomplishment was the production of all four stereoisomers **120**–**123** with high e.e. and d.e. using the two biocatalysts in a complementary process (see Scheme 37b). Later, the same group described two novel biocatalytic methods for the formal *anti*-Markovnikov hydroamination and hydration of alkenes (**124**) [67]; those methods utilized a one-pot cascade biotransformation and involved two different enzymatic cascades. For hydroamination, an epoxidation-isomerization-amination reaction sequence was established. Thus, an *E. coli* strain coexpressing styrene monooxygenase, styrene oxide isomerase, ω-transaminase and alanine dehydrogenase catalyzed the hydroamination of several aryl alkenes to produce the corresponding terminal amines **125** at high conversion rates and exclusive *anti*-Markovnikov selectivity. This reaction was performed using approximately 1 mg of substrate and resting *E. coli* cells. The reported substrate scope was wide, and styrene derivatives with substituents such as fluorine, chlorine and methoxy groups were converted to the corresponding phenethylamines **125** in high yields. Only the anti-Markovnikov products were produced; see Scheme 38a. A sequence of epoxidation-isomerization-reduction was developed for the hydration of aryl alkenes. As a result, another *E. coli* strain coexpressing styrene monooxygenase, styrene oxide isomerase, and phenyl-acetaldehyde reductase catalyzed the hydration of several aryl alkenes to the corresponding terminal alcohols (**126**) at high conversion rates and very high *anti*-Markovnikov selectivity. This process was conducted with resting cells of the biocatalyst. The scope of the hydration was evaluated with several substituted 2-phenylethanols. Fluoro-, methyl- and methoxy-substituted substrates were biotransformed at high conversion rates and with the exclusive production of the anti-Markovnikov alcohols (see Scheme 38b). In the same communication, the authors also reported the development of enantioselective hydroamination and hydration reactions; they chose compound **127** as model substrate and performed the enantioselective cascade epoxidation-isomerization for the synthesis of the corresponding chiral aldehyde **129** using the very same enzymes that were previously employed in the epoxidation-isomerization step. Once the enzymes achieved synthetic access to compound **129**, the enantioselective hydroamination was studied using several previously reported transaminases to catalyze the transamination step, producing the corresponding amine **130**. Thus, an engineered *E. coli* biocatalyst was tested for the enantioselective hydroamination of α-methylstyrene (**127**) in a cascade reaction, producing the corresponding amine (**130**) at an 84% conversion rate and a 92% e.e. On the other hand, a horse liver alcohol dehydrogenase was employed for the asymmetric hydration of compound **129**; thus, an engineered *E. coli* biocatalyst was designed and tested, affording the corresponding alcohol **131** at an 81% conversion rate and 97% e.e. (see Scheme 38c).

**Scheme 39:**
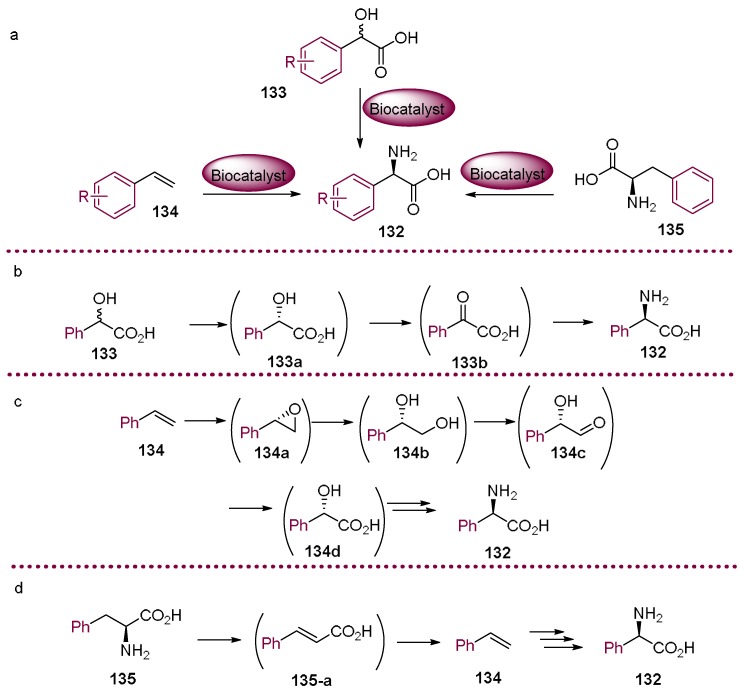
Cascade biotransformations. (**a**) synthesis of aminoacids; (**b**) synthesis of phenylglycine using mandelic acid; (**c**) synthesis of phenylglycine from styrene; (**d**) synthesis of phenylglycine from phenylalanine.

Subsequently, the researchers explored even more strategies [68], particularly for the synthesis of chiral amino acids. A strategy for the synthesis of d-phenylglycine derivatives **132** from the following three different starting materials was proposed: racemic mandelic acid (**133**), which is a cheap and easy available substrate; styrene (**134**), which is also a cheap hydrocarbon and under the biocatalytic conditions could be converted to the desired chemical without the use of cyanide; and l-phenylalanine (**135**), which is an easily available product of fermentation processes (Scheme 39a).

Each of these biologically based synthesis reactions is proposed to be performed in only one pot, avoiding the tedious and costly isolation of intermediates and waste generation. The researchers added the organic substrates in several portions, avoiding reach toxic concentrations of these substrates in the reaction media. This strategy also increased the scale of the synthesis reaction. The first process, the synthesis of d-phenylglycine-related compounds from racemic mandelic acid derivatives, was accomplished with >90% conversion in most cases using bio-engineered *E. coli* cells coexpressing (*S*)-mandelate dehydrogenase, mandelate racemase, d-phenylglycine aminotransferase and glutamate dehydrogenase. In some cases, the desired compounds were obtained after spontaneous precipitation from the reaction mixture; this procedure only uses O_2_, NH_3_ and glucose as inexpensive and green reagents and obtains the product as a single enantiomer on a scale of 200–800 mg; see Scheme 39b. For the second biotransformation, four additional enzymes, styrene monooxygenase, epoxide hydrolase, alcohol dehydrogenase and aldehyde dehydrogenase, were required to convert the styrene derivatives into the expected d-phenylglycine-related compounds using resting *E. coli* cells as biocatalyst. In most cases, high conversion rates (80–90%) and excellent e.e. (98–99%) were obtained on a scale of approximately 300 mg. See Scheme 39c. For the third process, resting *E. coli* cells coexpressing nine different enzymes were tested for their abilities to produce d-phenylglycine, and the synthetic utility of this approach was reported. The production of the desired amino acid as a single enantiomer achieved an 83% conversion rate on a 300 mg scale (see Scheme 39d).

## 7. Conclusions

Estimates of the number of organisms in the biosphere is a persistent challenge in biology, but in microbiology, this task is more complicated by the fact that the subjects of the census are only be viewed under the microscope and that further genetic analysis must be developed to undoubtedly identify a single species. Some estimates of the global number of bacterial species range from 10^5^ to 10^6^ [69]. The calculated number of fungal species is approximately 10^6^ [70]; on the other hand, the number of protist species is frequently estimated to range from 10^5^ to 10^6^ [71]. In this scenario, the number of potentially active biocatalysts and the possibilities of exploring this research field is enormous; organic chemists have understood this potential. Consequently, chemo-, regio- and stereoselective biotransformations using whole cells as biocatalysts have been reported continuously for decades, and an increasing number of these biotransformations have been published recently. The most important explanation for these advancements is that the use of whole cells as biocatalysts offers some relevant advantages with respect to either traditional chemical approaches and isolated enzyme preparations, such as milder reactions conditions, high selectivities and yields, the possibility of preparing one specific enantiomer, depending on the microorganism used, and the elimination of the need for expensive cofactors because the cell automatically supplies these cofactors, among others that might be mentioned. Additionally, recently, the collaboration between organic chemists and molecular biologists has enabled the use of specific enzymes overexpressed in easy-to-use microorganisms, such as *E. coli* and baker’s yeast, which substantially increases the future applications of this technology. A relevant disadvantage of using whole cells as biocatalysts is product isolation, which is occasionally cumbersome, and other enzymatic processes can occur simultaneously. Moreover, some relevant transformation reactions are still in development, and the scale for these processes must be increased to achieve a real impact on synthetic organic chemistry. In addition, for biotransformation reactions with pathogenic microorganisms, special conditions are required, and every single experiment should be performed under careful microbial control. Nevertheless, from our perspective, the advantages of this methodology outweigh the disadvantages. We hope that several research groups will continue exploring the opportunities of this exciting area of research and that readers will find this review useful in their research.
